# Ageing affects subtelomeric DNA methylation in blood cells from a large European population enrolled in the MARK-AGE study

**DOI:** 10.1007/s11357-021-00347-9

**Published:** 2021-04-19

**Authors:** Maria Giulia Bacalini, Anna Reale, Marco Malavolta, Fabio Ciccarone, María Moreno-Villanueva, Martijn E. T. Dollé, Eugène Jansen, Tilman Grune, Efstathios S. Gonos, Christiane Schön, Jürgen Bernhardt, Beatrix Grubeck-Loebenstein, Ewa Sikora, Olivier Toussaint, Florence Debacq-Chainiaux, Miriam Capri, Antti Hervonen, Mikko Hurme, P. Eline Slagboom, Nicolle Breusing, Valentina Aversano, Stefano Tagliatesta, Claudio Franceschi, Maria A. Blasco, Alexander Bürkle, Paola Caiafa, Michele Zampieri

**Affiliations:** 1 IRCCS Istituto delle Scienze Neurologiche di Bologna, Bologna, Italy; 2 Department of Experimental Medicine, Sapienza University of Rome, Viale Regina Elena 324, 00161 Rome, Italy; 3 Advanced Technology Center for Aging Research, IRCCS INRCA, 60121 Ancona, Italy; 4 IRCCS San Raffaele Pisana, Department of Human Sciences and Promotion of the Quality of Life, San Raffaele Roma Open University, 00166 Rome, Italy; 5 Molecular Toxicology Group, Department of Biology, University of Konstanz, 78457 Konstanz, Germany; 6 Human Performance Research Centre, Department of Sport Science, University of Konstanz, 78457 Konstanz, Germany; 7 Centre for Health Protection, National Institute for Public Health and the Environment, PO Box 1, 3720 BA Bilthoven, The Netherlands; 8 Department of Molecular Toxicology, German Institute of Human Nutrition Potsdam-Rehbruecke (DIfE), 14558 Nuthetal, Germany; 9 NutriAct-Competence Cluster Nutrition Research Berlin-Potsdam, 14458 Nuthetal, Germany; 10 Institute of Biology, Medicinal Chemistry and Biotechnology, National Hellenic Research Foundation, Athens, Greece; 11 BioTeSys GmbH, Schelztorstr. 54-56, 73728 Esslingen, Germany; 12 Research Institute for Biomedical Aging Research, University of Innsbruck, Rennweg, 10, 6020 Innsbruck, Austria; 13 Laboratory of the Molecular Bases of Ageing, Nencki Institute of Experimental Biology, Polish Academy of Sciences, 3 Pasteur street, 02-093 Warsaw, Poland; 14 URBC-NARILIS, University of Namur, Rue de Bruxelles, 61, Namur, Belgium; 15 DIMES- Department of Experimental, Diagnostic and Specialty Medicine, Alma Mater Studiorum- University of Bologna, 40126 Bologna, Italy; 16 Faculty of Medicine and Health Technology, Tampere University, FIN-33014 Tampere, Finland; 17 Department of Molecular Epidemiology, Leiden University Medical Centre, Leiden, The Netherlands; 18 Department of Applied Nutritional Science/Dietetics, Institute of Nutritional Medicine, University of Hohenheim, 70599 Stuttgart, Germany; 19 Laboratory of Systems Medicine of Healthy Aging and Department of Applied Mathematics, Lobachevsky University, Nizhny Novgorod, Russia; 20 Telomeres and Telomerase Group, Spanish National Cancer Research Centre (CNIO), Madrid, Spain; 21 Department of Cellular Biotechnologies and Haematology, Sapienza University of Rome, Viale, Regina Elena 324, 00161 Rome, Italy

**Keywords:** Ageing, Subtelomere, Epigenetics, DNA methylation, Down syndrome, Centenarian offspring

## Abstract

Ageing leaves characteristic traces in the DNA methylation make-up of the genome. However, the importance of DNA methylation in ageing remains unclear. The study of subtelomeric regions could give promising insights into this issue. Previously reported associations between susceptibility to age-related diseases and epigenetic instability at subtelomeres suggest that the DNA methylation profile of subtelomeres undergoes remodelling during ageing. In the present work, this hypothesis has been tested in the context of the European large-scale project MARK-AGE. In this cross-sectional study, we profiled the DNA methylation of chromosomes 5 and 21 subtelomeres, in more than 2000 age-stratified women and men recruited in eight European countries. The study included individuals from the general population as well as the offspring of nonagenarians and Down syndrome subjects, who served as putative models of delayed and accelerated ageing, respectively. Significant linear changes of subtelomeric DNA methylation with increasing age were detected in the general population, indicating that subtelomeric DNA methylation changes are typical signs of ageing. Data also show that, compared to the general population, the dynamics of age-related DNA methylation changes are attenuated in the offspring of centenarian, while they accelerate in Down syndrome individuals. This result suggests that subtelomeric DNA methylation changes reflect the rate of ageing progression. We next attempted to trace the age-related changes of subtelomeric methylation back to the influence of diverse variables associated with methylation variations in the population, including demographics, dietary/health habits and clinical parameters. Results indicate that the effects of age on subtelomeric DNA methylation are mostly independent of all other variables evaluated.

**Supplementary Information:**

The online version contains supplementary material available at 10.1007/s11357-021-00347-9.

## Introduction

Subtelomeres are transition DNA regions, ranging in size from 10 to 300 kb, which are found between chromosome-specific sequences and the telomeric repeats. Subtelomeres are poor in unique DNA sequences and genes. Instead, they are predominantly made up of a complex “patchwork” of blocks of sequences repeated in low copy numbers and characterised by a good intra- and inter-chromosomal sequence preservation [1–3]. Subtelomeric domains are considered heterochromatic regions, similar to telomeres. For instance, they contain HP1 protein and display histone modifications, such as H4K20me3 and H3K9me3, which are typical of heterochromatin. However, in contrast to the telomeres, the subtelomeres contain highly repetitive CpG dinucleotides, which make them prone to epigenetic regulation via DNA methylation [4, 5]. Telomere length is strongly affected by telomeric and subtelomeric chromatin modifications (reviewed in [4]). Subtelomeres tend to be strongly methylated both in humans and mice, and telomere elongation has been shown to be associated with subtelomeric hypomethylation, either following knockout of DNA methyltransferase enzymes [6] or in pathological conditions like cancer [7].

DNA methylation is among the epigenetic mechanisms that allow integration of intrinsic and environmental cues to shape genome functions [8]. Multiple interconnected pathways transduce these signals to the DNA methylation machinery, which can modify the cytosine base by the action of the DNA methyltransferase enzymes (DNMT1 or DNMT3A/B) to form 5-methylcytosine (5mC). 5mC, in turn, can be iteratively modified by the ten-eleven translocation (TET) family of proteins (TET1, TET2 and TET3) to produce the oxidation products 5-hydroxymethylcytosine (5hmC), 5-formylcytosine (5fC) and 5-carboxylcytosine (5caC). These 5mC oxidation products can act as intermediary compounds in the conversion of 5mC to unmodified cytosine, thus providing the first steps in the pathway for active DNA demethylation [9].

Rearrangements in the epigenetic landscape of the genome are recognized as being one of the major hallmarks of the ageing process [10], and the study of age-related DNA methylation changes is conceivably a promising way towards enhancing our understanding of the molecular mechanisms of ageing [11, 12]. While the bulk of the genome encounters a global loss of DNA methylation during ageing, a subset of CpG sites displays age-associated DNA methylation changes that are highly reproducible across individuals [11, 13]. This finding allowed the development of DNA methylation-based predictors of age, termed epigenetic clocks. First-generation epigenetic clocks were built as predictors of chronological age. However, it has been shown that the improved precision of epigenetic clock estimates was at the expense of their ability to detect departures from the physiological trajectories of ageing, possibly associated with age-related phenotypes and diseases [14, 15]. Therefore, attention has recently been devoted also to those CpG sites that do not necessarily display strong associations with chronological age but nevertheless are informative of the deviation of biological age from chronological age [13, 16].

In particular, the role of subtelomeric DNA methylation in ageing and age-related diseases is attracting an increasing level of interest [17]. Buxton et al. demonstrated that DNA methylation of subtelomeric sequences is associated with telomere length [18]. The mechanisms underlying the regulation of subtelomeric DNA methylation and its functional implications are yet to be fully understood. However, some previous reports have demonstrated the correlation between the subtelomeric DNA methylation and the development of neurodegenerative disorders (i.e. Alzheimer’s disease and Parkinson’s disease) [19–22], metabolic disorders (i.e. diabetes) [23] and some sporadic malignancies [7, 24–28]. Since these diseases are typically associated with ageing, these observations have led to the hypothesis that alterations in the *status* of subtelomeric methylation might be related to the ageing process.

This hypothesis is also supported by some initial evidence of an age-related change of subtelomeric DNA methylation in the white blood cells of healthy Japanese individuals [29]. However, the relationship between subtelomeric DNA methylation and ageing has not been investigated in large human populations so far.

Based on these premises, the present study has tested the association of subtelomeric methylation with ageing in the context of a large-scale European study, as part of the MARK-AGE project. MARK-AGE is a Europe-wide population study, supported by the European Commission (FP7), aiming to discover new biomarkers of ageing [30, 31]. MARK-AGE is mainly a cross-sectional study, in which age-stratified individuals (age range 35–75 years) were randomly recruited between 2008 and 2013 in seven European countries. The MARK-AGE study is largely representative of the general population, as the exclusion criteria included only seropositivity for HIV, HCV and HBV (except for seropositivity by vaccination) and the presence of actual cancer/current use of anti-cancer drugs or glucocorticoids. Furthermore, subjects born from a long-living parent belonging to a family with long-living sibling(s) and persons with progeroid syndromes were included as models of successful and unsuccessful ageing, respectively. Anthropometric, clinical and social data have been collected in a standardised manner, and a wide range of potential biomarkers of age was tested.

Here, we assessed the DNA methylation *status* of two subtelomeric regions. We analysed their variation with age in peripheral blood mononuclear cells (PBMC) from more than two thousand age-stratified donors (35–75 years), representative of the general population of eight European countries [31].

Our results indicate that age indeed influences the methylation level and patterns of the subtelomeres in PBMC. This association did not significantly depend on several nutritional, lifestyle or clinical variables, which influence subtelomeric DNA methylation in the population.

This study also took into consideration subtelomeric DNA methylation in models of successful (subjects born from a long-lived parent) and unsuccessful (subjects affected by Down syndrome, DS) ageing [31].

The study of these two subgroups provided further validation of the relationship between subtelomeric methylation and ageing, as the age-related methylation dynamic of the subtelomeres was consistent with their putative divergent rate of ageing.

## Methods

### Study design, recruitment, data and blood cell collection

The details of the MARK-AGE project have been the subject of previous publications, to which we refer for the study design [30, 31], data collection (i.e. demographic, anthropometric, clinical and social data) [32], the standard operating procedures (i.e. subject recruitment, collection, shipment and distribution of biological samples) [33] and management and processing of the MARK-AGE database [34, 35].

PBMC isolation procedure has been previously described [33, 36]. Briefly, the PBMC were isolated from EDTA whole blood, obtained by phlebotomy after overnight fasting, by discontinuous density gradient centrifugation in Percoll and subsequently cryopreserved to allow shipment, distribution and storage of the samples until analysis.

### DNA extraction

The PBMC samples were thawed by incubation at 37 °C, followed by dropwise addition of RPMI containing 10% FCS to a final dilution of 1:20. Cells were collected by centrifugation and processed for DNA extraction.

Isolation of total DNA was performed using the QIAamp 96 DNA Blood Kit (Qiagen, Hilden, Germany) according to the manufacturer’s instructions. DNA concentration, purity and integrity were evaluated as previously described [37].

### Quantitative DNA methylation analysis

The EpiTYPER assay (Sequenom, San Diego, USA) [38] was used to quantitatively assess the DNA methylation of two subtelomeric regions located in the short arm of chromosome 5 (5p; chr5:12,038–12,237 in the GRCh37/hg19 assembly) and in the long arm of chromosome 21 (21q), respectively. DNA (1 μg) was bisulphite-converted using the EZ-96 DNA Methylation Kit (Zymo Research, Irvine, USA) with the following modifications: incubation in CT buffer for 21 cycles of 15 min at 55 °C and 30 s at 95 °C, elution of bisulphite-treated DNA in 100 μl of water. Bisulphite-specific primers were designed using the EpiDesigner software (https://www.epidesigner.com) and checked by SYBR Green-based melt curve to evaluate the specificity of the amplification. The selected primers amplified the following regions, annotated in the GRCh37/hg19 assembly: chr5:12,038–12,237 (5p Fw: aggaagagagTTTTTTTTATTATAGATGTTGGGGG; 5p Rv: cagtaatacgactcactatagggagaaggctCCCAAACCTTCCTTAAAAACATCT); chr21:48,081,403-48,081,721: 21q Fw: aggaagagagGTTTTGTTGTGGAAAGGTTTAGTT; 21q Rv: cagtaatacgactcactatagggagaaggctCCCTCAAAATCTAAATCAAACAAAT. PCR was performed on 10 ng of converted DNA.

### Statistical analysis

Batch effect correction of methylation data was performed via the Partek Genomic Suite 6.6 implemented with the ANOVA tool (Partek Incorporated). We considered each EpiTYPER experimental plate (containing about 100 samples with study groups not evenly distributed across plates) as a batch.

For continuous variables, normal distribution of data was verified by the Kolmogorov–Smirnov and Shapiro–Wilk normality tests (data not shown) as previously described [36]. Identification and handling of outliers were performed as previously described [36].

The characteristics of the study population across age groups were analysed by the one-way-ANOVA (continuous variables) or chi-square test (categorical variables).

Associations between variables have been analysed by both parametric and non-parametric tests. The generalised linear model (GLM) method was used as a parametric approach for comparisons between groups. The GLM was also used to investigate the influence of confounding variables (tested as categorised and continuous variables). The Kruskal–Wallis (KW) method was used as a non-parametric approach to compare between groups. When a significant *p* value was found, pairwise comparisons (adjusted for multiple comparisons by Bonferroni’s and Dunn’s methods for the GLM and the KW tests, respectively) were used to identify significant differences between groups. The GLM and KW test included categorical and categorised continuous variables as predictors. Associations between continuous variables were also investigated by linear regression using both parametric (Pearson) and non-parametric (Spearman) correlations. Correlations were additionally investigated by stratified bootstrap sampling (1000 bootstrap samples). Effect sizes in the GLM and KW tests were estimated by the eta square (*η*^2^) and epsilon square (*ε*^2^) indexes (*η*^2^ values ≅ 0.01, ≅ 0.06 and > 0.14 indicate weak, moderate and strong effect sizes, respectively; *ε*^2^ values <0.04, <0.16, <0.36 and > 0.36 indicate weak, moderate, relatively strong and strong effect sizes, respectively).

For the generation of the Methylation Age of SubTelomere index (MAST), we adopted a strategy based on regression analysis for multiple biomarkers. Briefly, methylation levels of all CpG sites were combined by linear regression using age as dependent variable in the randomly recruited age-stratified individuals from the general population (RASIG). MAST was then calculated in GO, SGO and DS as the sum of the methylation levels of each CpG site multiplied by its corresponding coefficient derived from RASIG (MAST = Constant + ∑ linear regression coefficient of methylation level of CpG × methylation level of CpG).

All statistical analyses were carried out using SPSS software (IBM SPSS Statistics Version 23.0, New York, USA).

## Results

### Study population

The population under study consisted of 3155 individuals divided into three groups (Table 1). The largest group, representative of the “average” ageing population, consisted of randomly recruited age-stratified individuals from the general population (RASIG) of seven European countries covering the age range of 35–75 years. Stratification of RASIG into four consecutive 10-year age groups guaranteed an almost homogenous distribution of sample size, a balanced sex ratio and a broad area of origins across the whole age range studied.

**Table 1:** Study population

Subject group	RASIG						GO				SGO				DS
Age group	All	35	45	55	65	*p*	All	55	65	*p*	All	55	65	*p*	
Age range (years)	35–75	35–44	45–54	55–64	65–75		55–75	55–64	65–75		55–75	55–64	65–75		19–68
*N*	2308	494	576	628	610		512	253	259		283	155	128		52
Age (year)	55.9 ± 113	40.0 ± 2.8	50.1 ± 2.8	60.2 ± 2.9	69.9 ± 3.1	**˂ 0.001**	64.7 ± 4.8	60.6 ± 2.7	68.7 ± 2.6	**˂ 0.001**	64.2 ± 4.6	60.8 ± 2.5	68.6 ± 2.6	**˂ 0.001**	40.2 ± 12.2
Male % (*n*)	48.5 (1119)	47.9 (237)	47.4 (273)	49.5 (311)	48.8 (298)	0.89	42.6 (218)	43.9 (111)	41.3 (107)	0.558	51.9 (147)	39.3 (61)	67.2 (86)	**˂ 0.001**	53.84 (28)
BMI (kg/m^2^)	26.2 ± 4.5	25.1 ± 4.6	25.6 ± 4.1	26.8 ± 4.7	27.1 ± 4.3	**˂ 0.001**	26.6 ± 4.3	26.6 ± 4.4	26.6 ± 4.2	0.165	27.4 ± 4.4	27.3 ± 4.5	27.6 ± 4.2	0.642	
˂ 25	44.8 (1032)	57.3 (283)	50.2 (289)	40.7 (255)	33.8 (205)		22.7 ± 1.7	22.4 ± 1.9	22.9 ± 1.4		22.7 ± 1.5	22.6 ± 1.4	22.8 ± 1.6		
25 to ˂ 30	37.9 (872)	30.4 (150)	35.9 (207)	38.2 (239)	45.5 (276)		27.0 ± 1.3	27.0 ± 1.4	27.1 ± 1.2		27.2 ± 1.3	27.1 ± 1.3	27.2 ± 1.3		
≥ 30	17.3 (399)	12.3 (61)	13.9 (80)	21.1 (132)	20.8 (126)		32.9 ± 3.6	32.7 ± 3.5	33.1 ± 3.7		33.7 ± 2.9	33.8 ± 3.0	33.4 ± 2.8		
Austria	17.3 (399)	20.0 (99)	17.4 (100)	16.4 (103)	15.9 (97)	**˂ 0.001**				0.092				**0.012**	
Belgium	11.4 (262)	6.5 (32)	12.0 (69)	12.7 (80)	13.3 (81)		15.4 (79)	17.8 (45)	13.1 (34)		12.0 (34)	12.3 (19)	11.7 (15)		
Finland	4.0 (93)	1.8 (9)	2.1 (12)	5.6 (35)	6.1 (37)		26.2 (134)	24.1 (61)	28.2 (73)		18.4 (52)	18.1 (28)	18.7 (24)		
Germany	15.5 (358)	12.8 (63)	16.8 (97)	15.8 (99)	16.2 (99)										
Greece	17.0 (392)	19.2 (95)	18.6 (107)	15.6 (98)	15.1 (92)		3.5 (18)	4.7 (12)	2.3 (6)		1.8 (5)	0.6 (1)	3.1 (4)		
Italy	17.2 (398)	19.8 (98)	17.2 (99)	15.9 (100)	16.6 (101)		18.4 (94)	17.4 (44)	19.3 (50)		19.4 (55)	23.9 (37)	14.1 (18)		100 (52)
Poland	17.6 (406)	19.8 (98)	16.0 (92)	18.0 (113)	16.9 (103)		13.7 (70)	16.2 (41)	11.2 (29)		14.8 (42)	18.7 (29)	10.2 (13)		
The Netherlands							22.9 (117)	19.8 (50)	25.9 (67)		33.6 (95)	26.4 (41)	42.2 (54)		

Values are mean ± SD for continuous variables and percentage (number) for categorical variables

*p* value: one-way ANOVA (continuous variables) and chi-square test (prevalence, for categorical variables). Bold text indicates a statistically significant difference ( *p* value < 0.05). Definition of abbreviations is provided in the supplementary list

A second group represented a presumed model of successful or “retarded” ageing. It consisted of the offspring of nonagenarians, allegedly predisposed to longevity because of their genetic background. This group has been previously studied in the framework of the GEHA (Genetics of Healthy Ageing) project [39] and is hereafter referred to as GO (GEHA offspring). To check for lifestyle and environmental effects, the GO group was compared to the group of their spouses, which was named the SGO (spouses of the GO) [31] group. Both groups covered the age range of 55–75 years.

Finally, subjects affected by DS, a known segmental progeroid syndrome, were considered as a model of premature/accelerated ageing [31].

Being composed of quite rare individuals, the GO, SGO and DS groups show limited sample size, a not wholly balanced sex ratio in some age groups and a limited geographical distribution of sample origin.

The body mass index (BMI) appeared to increase with age in RASIG and is consistently higher in SGO compared to GO, indicating that the analysed population was effectively representative of a physiological ageing process.

### Characteristics of the analysed subtelomeric regions

**Fig. 1:**
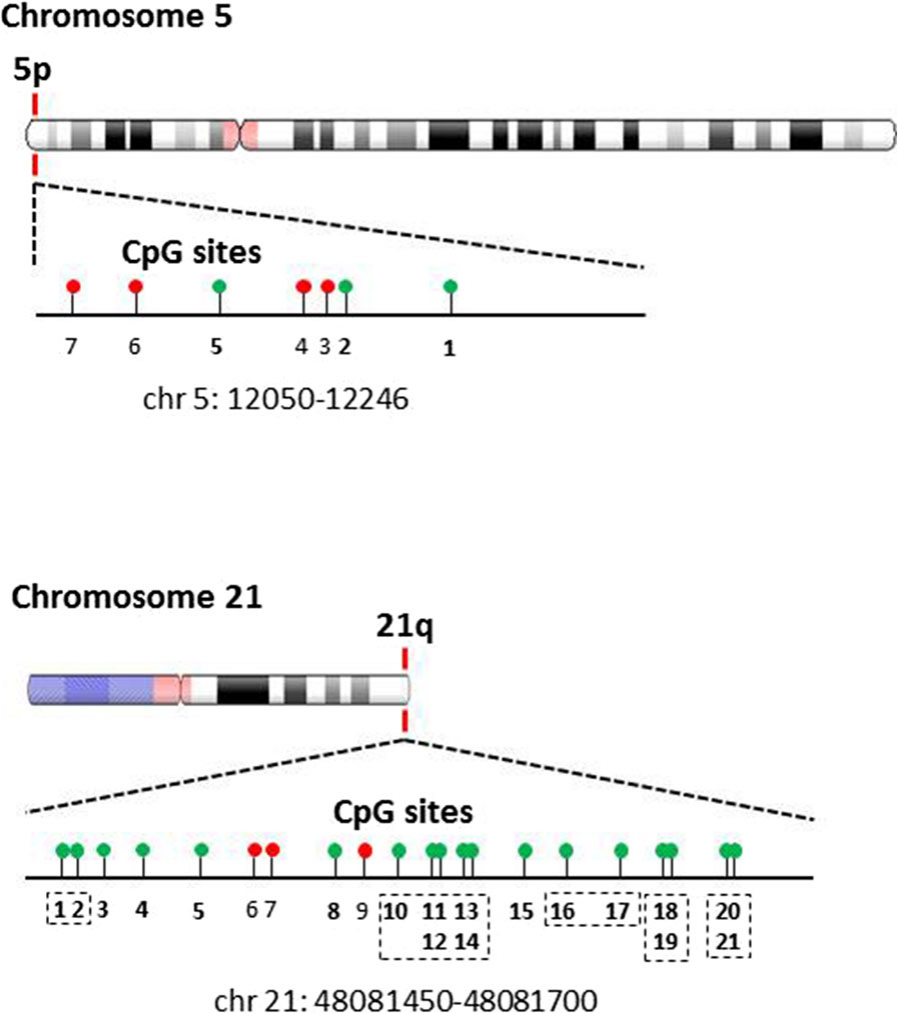
CpG distribution of the 5p and 21q subtelomeric regions. Schematic representation of CpG site distribution within the analysed regions. Green and red circles represent the analysed and non-analysed CpG sites, respectively. The dashed boxes indicate the CpG sites that were measured as single methylation units in the EpiTYPER assay. The genomic positions refer to the 2009 (GRCh37/hg19) assembly

Subtelomeric DNA methylation was quantified in PBMC-derived genomic DNA using the EpiTYPER assay. This assay measures the methylation percentage of single CpG sites or groups of adjacent sites, termed CpG units, included in a target genomic sequence. Two target sequences were selected among the subtelomeres that have low structural variation and lack sequence gaps and large duplications [40, 41]: the first one is located on the short arm of chromosome 5 (5p) and includes 3 detectable CpG sites; the second one is located in the long arm of chromosome 21 (21q) and includes 18 detectable CpG sites, grouped in 10 CpG units. The localisation of the target genomic sequences and the CpG sites covered by the analysis are shown in Fig. 1.

### Methylation changes of the subtelomeric CpG sites with age in the general population

The relationship between variation in subtelomeric methylation and physiological ageing was investigated in the RASIG group, representative of the general population in the chronological age range of 35–75 years.

**Fig. 2:**
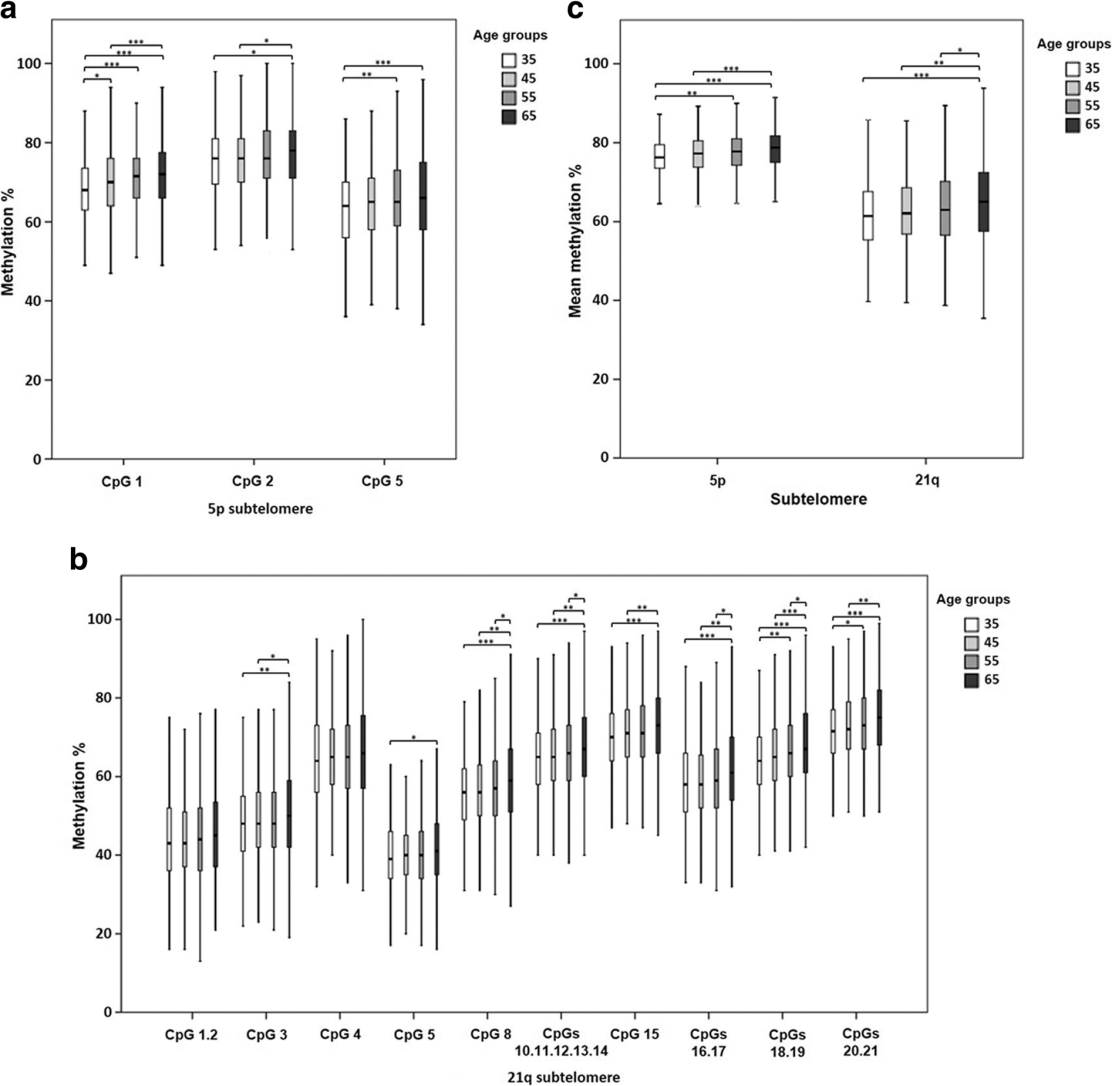
DNA methylation level of the 5p and 21q subtelomeres in RASIG stratified by age. **a** Methylation percentage of the CpG sites in the 5p subtelomeric region. **b** Methylation percentage of the CpG sites in the 21q subtelomeric region. **c** Mean methylation percentage calculated from all the CpG sites of the 5p and 21q subtelomeres. Data are depicted by box-and-whisker plots. The horizontal line indicates the median. The lower and the upper edge of the box show the first and the third quartile, respectively. The whiskers show the maximum and the minimum data values. Pairwise comparisons resulting in a significant Kruskal–Wallis test are indicated by the asterisks. *p < 0.05, **p < 0.01, ***p < 0.001

The association between CpG methylation level and age was investigated both by an age stratification approach and by linear correlation analysis. By comparing the 10-year age groups, we observed a progressive increase in methylation with increasing age of most of the CpG sites on both subtelomeres, as well as an increase of the mean subtelomeric methylation calculated in each individual (Supplementary Table S1). The significance of these associations was confirmed by both non-parametric (Kruskal–Wallis, KW) and parametric (generalised linear model, GLM) tests, the latter accounting for the effects of sex and recruitment centre as potential confounding factors. One exception was the 21q CpG unit 1.2 which was significantly associated with age only in the GLM. Pairwise comparison tests indicated that the differences mainly concerned the most extreme age groups. These data are shown graphically in Fig. 2.

**Fig. 3:**
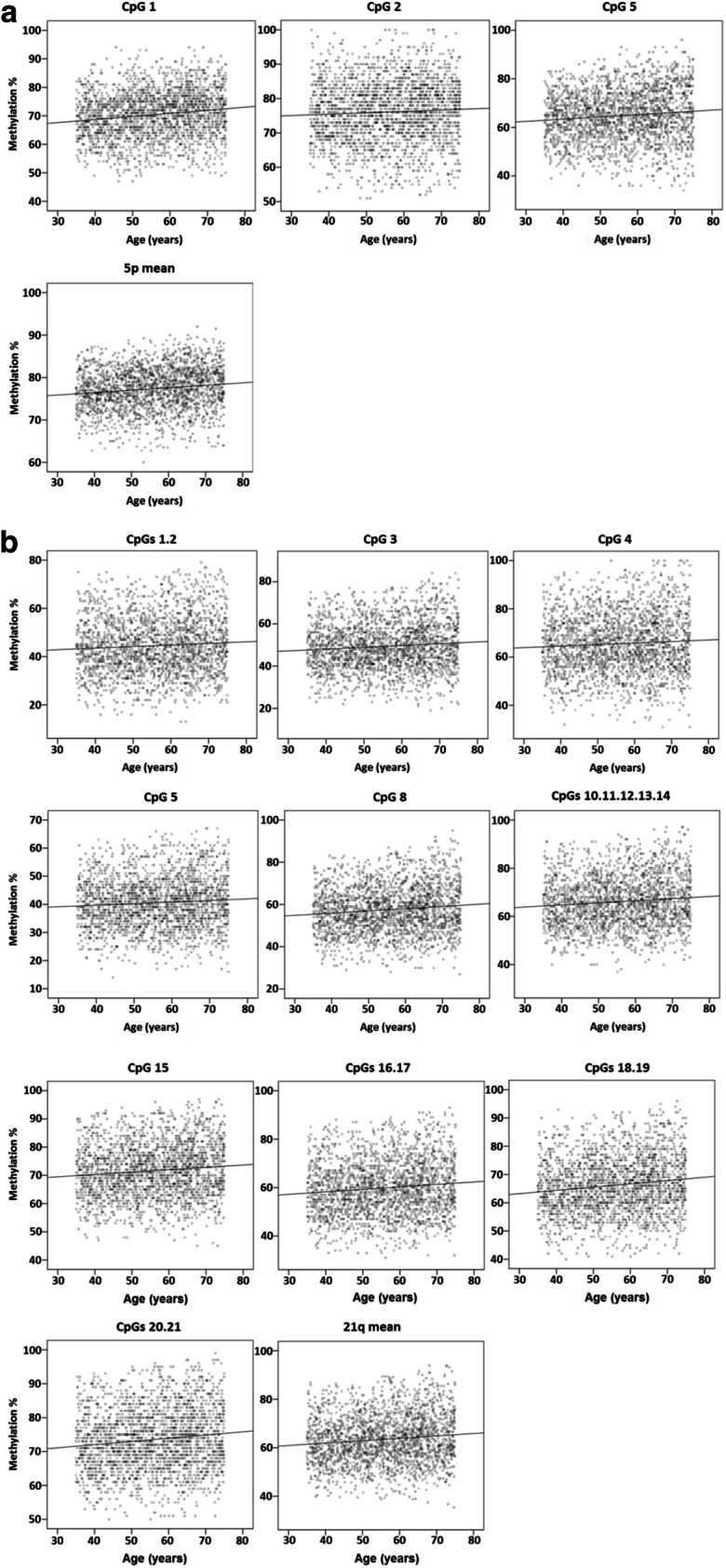
Correlation between the DNA methylation level of the 5p and 21q subtelomeres and age in RASIG. **a** CpG methylation percentage and average methylation percentage of the 5p subtelomere. **b** CpG methylation percentage and average methylation percentage of the 21q subtelomere. Data are depicted by scatterplots, including the line of best fit

All CpG sites showed a weak positive, albeit highly significant, linear correlation with age, both in non-parametric (Spearman) and parametric (Pearson) tests, which included a bootstrap stratified resampling in order to control for the effects of sex and recruitment centre (Supplementary Table S2 and Fig. 3).

### Generation of a cumulative epigenetic age predictor by combining the age-related DNA methylation changes on 5p and 21 q subtelomeric regions

Based on the highly concordant linear association of all the CpG sites with age, we chose to create a summary index that illustrates the cumulative importance of the DNA methylation of all sites in predicting age. Several multiple linear regression methods were tested in the RASIG population using the methylation value of all the 5p and 21q CpG sites as the predictor variables and age as the response variable (Supplementary Table S3, methods 1–8). Method 1 (linear regression based on enter method) outperformed all other methods in modelling the relationship between age and DNA methylation changes as it provided predicted values with the highest correlation vs age in both parametric (*R* = 0.176, *p* < 0.001) and non-parametric tests (*R* = 0.174 *p* < 0.001). Further details on this regression model are given in Supplementary Table S4. Notably, the coefficient of determination (*R* = 0.254) of the model was higher than the association with age of each of the CpG sites methylation in the binary correlation analyses (Supplementary Table S2). This indicated that the combined model was more informative for age prediction than the methylation values of each single CpG site. The linear predictor of the model was therefore used to combine the methylation status of all CpG sites into a single subtelomeric DNA methylation age index (from now on referred to as Methylation Age of SubTelomeres or MAST) for each individual. The linear regression coefficients, calculated from the RASIG group, were then used to compute MAST in all the other populations (GO, SGO, DS) included in this study.

Notably, given the relatively low coefficient of determination of the model, MAST should not be regarded as a DNA methylation-based predictor of age (in other words, MAST is *not* an epigenetic clock) but as a cumulative index measuring the combined age-related DNA methylation changes of the 5p and 21q subtelomeric regions.

### Association of MAST with age and telomere length in the general population

**Fig. 4:**
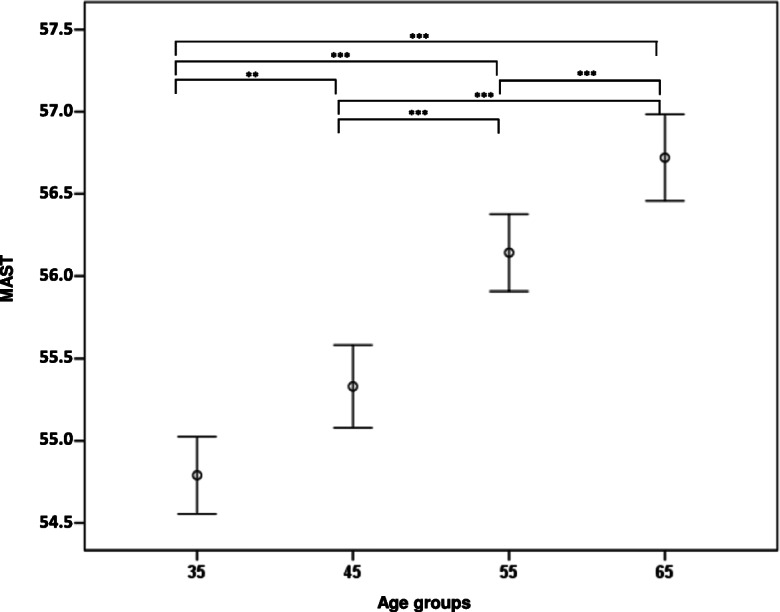
Level of MAST in RASIG stratified by age. Data are represented by mean ± S.D. Pairwise comparisons resulting in a significant GLM analysis, followed by Bonferroni post hoc test, are indicated by the asterisks. The GLM was adjusted for sex and recruitment centre. *p < 0.05, **p < 0.01, ***p < 0.001

Stratification analysis was used to further evaluate the association of MAST with age. As shown by a GLM test adjusted for sex and recruitment centre, a significant increase of MAST was detected with increasing age in RASIG (Table 2 and Fig. 4). Non-parametric tests confirmed this trend (Supplementary Table S5).

**Table 2:** GLM analysis of the methylation age of subtelomeres (MAST) by age in RASIG

Variables	Type III		
	Wald chi-square	df	Sig.
Age group	134.080	3	**< 0.001**
Sex	2.181	1	0.140
Recruitment centre	3.773	1	0.052

Model: MAST (*s*), response variable; age group (*o*), factor variable; sex (*n*) and recruitment centre (*n*) were included as covariates (*o*, ordinal variable; *n*, nominal variable; *s*, scale variable). *Type III*, type III sum of squares. Bold text indicates a statistically significant *p* value

In order to investigate the possible functional importance of subtelomeric DNA methylation, we next examined the relationship between MAST, telomere length and age. In fact, subtelomeric DNA methylation is known to be closely related to the control of telomere length, and telomere shortening is associated with the ageing process. A correlation analysis showed that the positive relationship between MAST and age was paralleled by an inverse association between telomere length and age (Supplementary Table S6). These findings indicated that the increase in MAST with age could be associated with telomere shortening. In fact, a significant, albeit very weak in magnitude, negative correlation between MAST and telomere length supported such a connection (Supplementary Table S6). The difference in the correlation coefficient of MAST with age from that reported in Supplementary Table S4 may be due to the limited number of subjects tested for the telomere length included in this last analysis.

### Analysis of MAST in the offspring of nonagenarians and in persons affected by Down syndrome

To evaluate the association between subtelomeric DNA methylation and ageing progression, MAST was analysed in the GO and DS groups as models of decelerated and accelerated ageing, respectively.

**Fig. 5:**
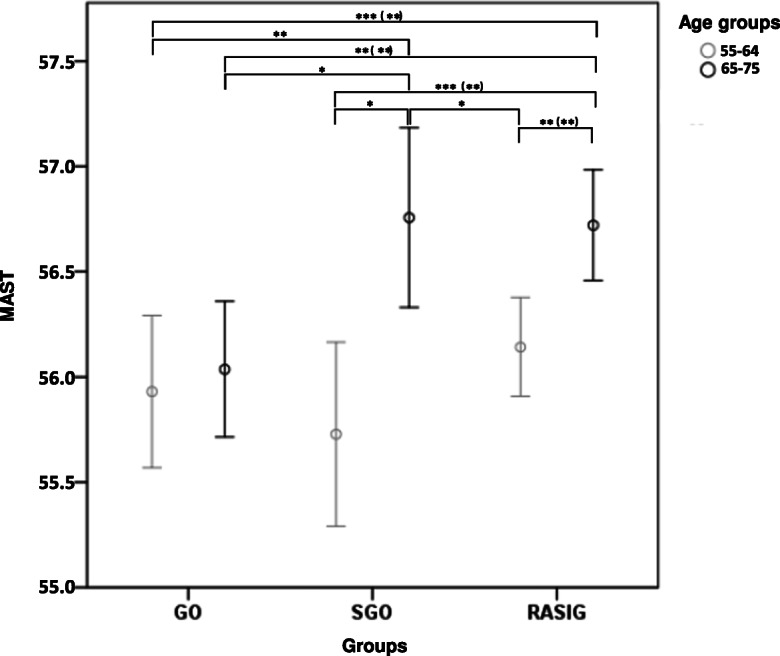
Level of MAST in GO vs SGO and RASIG stratified by age. Data are represented by mean ± S.D. Pairwise comparisons resulting in a significant GLM analysis, followed by least significant difference (and Bonferroni) post hoc tests, are indicated by the asterisks. The GLM was adjusted for sex and recruitment centre. *p < 0.05, **p < 0.01, ***p < 0.001

We tested if MAST was able to distinguish GO from SGO. Age-matched RASIG individuals (55–75 years) were used as an additional normal ageing control group. As reported in Fig. 5, significant differences between the two age subgroups (55–64 and 65–75) were found in the RASIG and in the SGO groups, but not in the GO group. Furthermore, in the 65–75 age group, MAST was significantly lower in GO compared to SGO and RASIG. A GLM test adjusted for sex and recruitment centre, as well as non-parametric comparison tests, confirmed this result (Table 3 and Supplementary Table S5).

**Table 3:** GLM analysis of MAST in GO vs SGO and RASIG in the population aged >54 years controlling for sex and recruitment centre

Variables	Type III		
	Wald chi-square	df	Sig.
Subject group (GO, SGO, RASIG)	8.943	2	**0.011**
Age group	15.199	1	**< 0.001**
Sex	0.658	1	0.417
Recruitment centre	0.524	1	0.469

Model: MAST (*s*), response variable; subject group (*n*), factor variable; age group (*o*), sex (*n*) and recruitment centre (*n*) were included as covariates (*o*, ordinal variable; *n*, nominal variable; *s*, scale variable). *Type III*, type III sum of squares. Bold text indicate a statistically significant *p* value

**Fig. 6:**
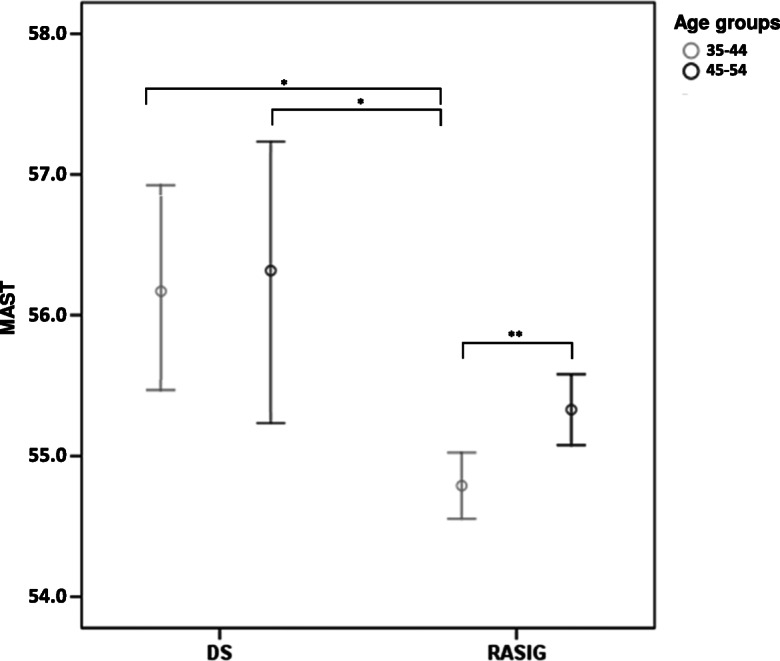
Level of MAST in DS vs RASIG stratified by age. Data are represented by mean ± S.D. Pairwise comparisons resulting in a significant GLM analysis, followed by least significant difference (and Bonferroni) post hoc tests, are indicated by the asterisks. The GLM was adjusted for sex and recruitment centre. *p < 0.05, **p < 0.01, ***p < 0.001

A similar analysis was performed on DS. Due to the limited sample size of the DS group, the analysis was limited to the age range of 35–54 years. Age- and recruitment centre–matched RASIG individuals were used as a control group. Again, significant differences in MAST between the two age subgroups (35–44 and 45–54) were found in the RASIG but not in the DS group (Fig. 6). MAST mean values in DS were significantly higher than those of 35–44-year-old RASIG. A GLM test adjusted for sex, as well as non-parametric comparison tests, confirmed this result (Table 4 and Supplementary Table S5).

**Table 4:** GLM analysis of MAST in DS vs RASIG in the age range of 35–55 years

Variables	Type III		
	Wald chi-square	df	Sig.
Subject group (DS, RASIG)	8.334	1	**0.004**
Age group	9.421	1	**0.002**
Sex	4.416	1	**0.036**

Model: MAST (*s*), response variable; subject group (*n*), factor variable; age group (*o*) and sex (*n*) were included as covariates (*o*, ordinal variable; *n*, nominal variable; *s*, scale variable). *Type III*, type III sum of squares. Bold text indicated a statistically significant *p* value

### Investigation of factors associated with subtelomeric DNA methylation changes in the general population

An extensive set of data on demographics, anthropometric and clinical parameters have been collected from RASIG, GO and SGO subjects enrolled in the MARK-AGE study [30]. A subset of these parameters, possibly implicated in DNA methylation and telomere homeostasis, was evaluated for its association with MAST changes in RASIG. These included demographic and anthropometric data (Supplementary Table S7), self-reported dietary and lifestyle habits (Supplementary Table S8), cardiovascular/diabetes risk factors (Supplementary Table S9) and expression of components of the DNA methylation molecular machinery in PBMC (Supplementary Table S10). Moreover, differential white blood cell count data were used to monitor the effect of changes in the PBMC sample composition (Supplementary Table S11). The analysis was performed by comparing MAST levels between RASIG individuals stratified into consecutive subgroups according to the variable of interest. Comparisons were performed by both non-parametric (KW) and parametric (GLM) tests. The latter allowed for confounding variables adjustment (i.e. sex and recruitment centre). The magnitude of the difference in MAST between subgroups (effect size) was estimated by calculating the *η*^2^ and the *ε*^2^ for the GLM and KW tests, respectively.

Several associations were identified by the KW test but were disproved by the GLM. These included the BMI, French fries and whole bread consumption, serum levels of glucose, total cholesterol and free fatty acids. The connection of these factors to MAST seemed, therefore, to be spurious and strongly influenced by confounding factors (i.e. sex and recruitment centre). Conversely, the association of MAST with glycated haemoglobin A1c was proved only by the GLM, which indicated that sex and recruitment centre were acting as positive confounders.

Based on the congruence between the KW and GLM tests, several factors appeared to be significantly associated with MAST.

Among demographics, the recruitment centre had an impact, evidently due to the samples recruited in Austria (Supplementary Table S7).

An influence of dietary/lifestyle habits on MAST was attributable to the consumption of white bread and alcoholic beverages (Supplementary Table S8). In both cases, increased MAST was detectable between the moderate- and high-consumption groups. In the case of white bread consumption, the GLM attenuated the significance of the association detected by the KW, indicating a partial confounding effect by sex and recruitment centre.

Regarding cardiovascular risk biomarkers (Supplementary Table S9), serum triglycerides, low-density lipoprotein cholesterol (LDL-C) and fibrinogen showed a significant positive association with MAST, although the effect of the confounding variables attenuated the association of LDL-C in the GLM analysis.

In the analysis of the association of MAST with the expression of enzymes involved in the methylation/demethylation processes (Supplementary Table S10), a significant association was found for DNMT1, DNMT3A and TET3. MAST increased in the upper quantiles of DNMT1 and DNMT3A expression, while it decreased in the case of TET3.

Among haematological parameters (Supplementary Table S11), MAST was associated with peripheral blood lymphocyte and neutrophil count and with the lymphocyte to monocyte count ratio. Differences mainly concerned the 1st vs the 4th quartiles. This association was positive for the lymphocyte count and the lymphocyte/monocyte count ratio, while it was negative for neutrophils count.

Most of the reported MAST associations showed weak effect sizes, except for TET3 and DNMT3A expression that showed moderate and relatively strong effect sizes, respectively.

### The contribution of selected variables on age-related changes in MAST in the RASIG and GO/SGO population

The variables found to be associated with MAST were next studied as potential causative factors in the association of MAST with age in RASIG and in its difference between the GO, SGO and RASIG groups.

Due to sample size limitations, data on DNMT3A, TET and TDG expression were excluded from the analyses.

The effect on the relationship between MAST and age in RASIG was examined by GLM analysis. The model tested MAST differences between the 10-year age strata, adjusted for all the factors that significantly affected MAST level as previously shown by both KW and GLM analyses (see Supplementary Tables S7–S11).

As shown in Table 5, none of the variables confounded the association of MAST with age groups, which, in fact, retained a highly significant *p* value (*p* < 0.001, see Table 2 for comparison) despite the inclusion of all the covariates. However, the DNMT1 transcript level and alcohol consumption seemed to have a certain weight in the model (Table 5).

**Table 5:** Contribution of selected variables and covariates on age-related changes of MAST in RASIG

Source	Type III		
	Wald chi-square	df	Sig.
Age group	72.680	3	**<0.001**
Recruitment centre	2.635	1	0.105
Sex	0.935	1	0.334
White bread consumption	0.032	1	0.858
Alcohol consumption	4.011	1	**0.045**
Triglycerides	1.825	1	0.177
LDL cholesterol	1.850	1	0.174
Lymphocytes	0.201	1	0.654
Lymphocyte to monocyte ratio	1.779	1	0.182
Neutrophils	0.552	1	0.45
Fibrinogen	3.604	1	0.058
Relative DNMT1 expression	4.578	1	**0.032**

Model: MAST (*s*), response variable; age group (*o*), factor variable; sex (*n*), recruitment centre (*n*), white bread consumption (*s*), alcohol consumption (*s*), triglycerides (*s*), LDL cholesterol (*s*), lymphocytes (*s*), ratio of lymphocyte to monocyte (*s*), neutrophils (*s*), fibrinogen (*s*) and DNMT1 expression (*s*) were included as covariates (*o*, ordinal variable; *n*, nominal variable; *s*, scale variable). *Type III*, type III sum of squares. Bold text indicates a statistically significant *p* value

Similar results were obtained by running several additional GLM models with the progressive inclusion of all the MAST-associated variables, categorised on the basis of having any significant association with MAST (i.e. also including variables showing significant interaction after the KW or GLM only) (Supplementary Table S12).

The crude models (models 1–4) included “standard” adjustment variables (recruitment centre, sex and lymphocyte to monocyte ratio). We next added to these crude models all the variables—one by one—associated with MAST either by both the GLM and KW tests (models 5–12) or by only one of the tests (models 14–19). The order in which the variables were added followed the criterion of obtaining the minimum reduction in the number of cases included in the model. Models 13, 20 and 21 accounted for the effect of all the potential confounding variables simultaneously. Models 20 and 21 differ for the inclusion of DNMT1 expression, which was the variable causing the greatest reduction in sample size.

No significant confounding effect on MAST variation across age groups was observed by any of the covariates. In fact, the association of MAST with age groups remained highly significant in all the tested models. Nevertheless, based on their significant *p* values, DNMT1, glucose, glycated haemoglobin and alcohol were found to be explanatory variables in the cumulative model (model 21) that contained all the other factors.

Conversely, the significance showed by other covariates (e.g. recruitment centre, sex, BMI, fibrinogen and LDL-C) in some of the models was dependent on the effect of other adjusting variables. In fact, their *p* value became non-significant in the cumulative models.

The same analyses were performed to test the variation of MAST between GO, SGO and RASIG.

The confounding effect of the factors associated with MAST in both the KW and GLM tests was negligible, both in the cumulative (Table 6) and in the stepwise analysis (Supplementary Table S13, models 1–14). The expression of DNMT1 and the lymphocyte to monocyte ratio were the covariates partially affecting MAST differences across subject groups (Table 6 and Supplementary Table S13, model 14). However, the significance of the association between MAST and subject groups was partially affected by the combination of all factors rather than by a single one (see the subject group *p* value in models 1–13 with respect to model 14 in the Supplementary Table S13).

**Table 6:** Contribution of selected variables and covariates on group-related (GO, SGO and RASIG) changes of MAST in individuals aged >54 years

Source	Type III		
	Wald chi-square	df	Sig.
Subject group (GO, SGO, RASIG)	8.485	2	**0.014**
Age	12.077	1	**0.001**
Recruitment centre	0.153	1	0.695
Sex	0.518	1	0.472
Alcohol consumption	1.345	1	0.246
White bread consumption	0.013	1	0.909
Triglycerides	0.019	1	0.890
LDL cholesterol	0.962	1	0.327
Fibrinogen	2.584	1	0.108
Lymphocytes	0.344	1	0.557
Lymphocyte to monocyte ratio	4.671	1	**0.031**
Neutrophils	0.358	1	0.550
Relative DNMT1 expression	4.369	1	**0.037**

Model: MAST (*s*), response variable; subject group (*o*), factor variable; age (*s*), sex (*n*), recruitment centre (*n*), white bread consumption (*s*), alcohol consumption (*s*), triglycerides (*s*), LDL cholesterol (*s*), lymphocytes (*s*), ratio of lymphocyte to monocyte (*s*), neutrophils (*s*), fibrinogen (*s*) and DNMT1 expression (*s*) were included as covariates (*o*, ordinal variable; *n*, nominal variable; *s*, scale variable). *Type III*, type III sum of squares

Similar conclusions could be drawn from the analysis of factors associated with MAST by the KW or the GLM tests. Each additional factor was, by itself, ineffective in explaining MAST association with subject groups (Supplementary Table S13, models 15–20), but the inclusion of all factors made this relationship not significant (*p* value = 0.065 in model 22).

The loss of significance of subject groups seemed to be driven by two factors: (i) a drop in the sample size when DNMT1 expression was entered in the model and/or (ii) to a synergy between DNMT1 expression and other variables, such as the lymphocyte/monocyte ratio (see model 21 vs model 22).

After the removal of DNMT1, the association of MAST with the subject groups regained a borderline significance (*p* value = 0.045 in model 21). Compared to previous models, the decrease in significance for the subject groups in this model could be due to the confounding effect of BMI, glycated haemoglobin, French fries and glucose, as well as to a smaller sample size.

## Discussion

Previous studies indicate the possibility that epigenetic changes occur at subtelomeric DNA regions in both physiological and abnormal ageing [19–23, 29, 42]. Here, this hypothesis has been tested in the context of a large-scale population-based study, which also provides a reference framework for factors that are associated with subtelomeric DNA methylation variation in the general population including demographics, clinical laboratory parameters, dietary and health habits. Furthermore, this study adopts the innovative approach of validating the identified putative subtelomeric methylation signature of ageing in highly informative groups represented by offspring of nonagenarians and subjects affected by Down syndrome, representing models of healthy ageing [43] and premature/accelerated ageing [44], respectively.

The method adopted for methylation analysis (EpiTYPER [38]) enabled us to get a quantitative measure of subtelomeric DNA methylation in blood mononucleated cells, largely at single-nucleotide resolution. Data obtained provided an unprecedented opportunity to systematically analyse the subtelomeric methylation profile and its variation with age in the general population.

The CpGs showed an intermediate level of methylation possibly reflecting an epigenetic heterogeneity across principal immune cells that form the PBMC population. However, this heterogeneity may also conform to variation within cell types, since varying degrees of subtelomeric methylation were reported in other studies even in cultured primary cells [45].

Nevertheless, each CpG site showed a limited range of methylation variation, thus forming a methylation pattern that appeared to be specific to the subtelomeric region. The methylation profile of subtelomeres seems therefore to be controlled by specific mechanisms and might reflect certain functions as it has recently been proposed for other regions showing intermediate methylation states in the human genome [46]. CpG methylation levels increased with age at almost all the assessed CpG sites. Moreover, the rate of increase of methylation with age seems to be similar across the different CpG sites. Consequently, the average methylation of the entire subtelomeric region increases without significant changes in its variability. This suggests that age-related hypermethylation of subtelomeres does not evolve randomly, unlike many other age-associated differentially methylated regions, where ageing is associated with an entropic decay of the epigenetic profile [47].

Overall, these findings contrast with previous results showing an age-related hypomethylation of global subtelomeric regions in blood from healthy individuals [29] and patients affected by age-related diseases such as Parkinson’s disease [21]. There are several possible explanations for this discrepancy. Firstly, compared to Maeda et al. [29], our cohort of cases was significantly more extensive and representative of a different and more heterogeneous geographical area. Secondly, we adopted an analytical method that has an advantage over the methylation-sensitive Southern blot assay used by Maeda et al., both in terms of quantitative accuracy and the number of CpG sites that can be assessed individually. Finally, we focused on two specific subtelomeric regions, while Maeda et al. evaluated the global methylation *status* of subtelomeres. This last point is of particular relevance since the methylation *status* of a subtelomeric region and its variation with age may depend on its specific chromosomal context, including the length of the adjacent telomere end. It is, therefore, possible that the increase in methylation with age reported here for 5p and 21q subtelomeric regions reflects their specific chromosomal context and represents the trend of a specific subset of the telomeres.

DNA methylation appears to change at a constant rate across age groups, indicating a linear relationship with chronological age. Regression analyses actually show a significant direct correlation between age and methylation level for all CpG sites. However, the proportion of methylation variation explained by age was small for every single site. By contrast, a subtelomeric DNA methylation index (MAST) based on the aggregate methylation *status* of all CpG sites provided a better fit with age and outperformed every single site in discriminating between age groups. Biologically, this suggests that the amount that each CpG methylation contributes to age is additive and/or that additional information may come from the effects of age on the DNA methylation pattern of the whole subtelomeric region.

This point should also be taken into account when interpreting the negative correlation between MAST and the average length of the bulk telomere population shown in Supplementary Table S6. These results are consistent with previous observations linking a loss of subtelomeric methylation to telomere elongation [6, 7] and suggest that subtelomeric DNA hypermethylation is related to telomere shortening in ageing. However, this association might not involve all of the subtelomeres. In fact, it has been suggested that the correlation between subtelomeric methylation and telomere length is variable, in terms of both strength and direction, across different chromosomes and/or different regions of the same subtelomere [27]. This insight could also offer a plausible explanation for the observation that the amount of the telomere length variation explained by MAST is relatively small.

Remarkably, MAST was more correlated with age than with telomere length. This finding suggests that part of the DNA methylation ‘signature’ of age in subtelomeres is not related to telomere length control. DNA methylation could also affect the expression of genes related to ageing via the telomere position effect (TPE) [48] and/or by controlling transcription of the non-coding telomeric RNAs (TERRA), recently described as trans-acting epigenetic modulators of multiple genes throughout the genome [49].

The trend of MAST in the GO and DS with respect to RASIG further supports a link of subtelomeric methylation and the ageing process. Higher MAST in DS is compatible with the accelerated ageing phenotype of this syndrome and mirrors previous data indicating that telomere length maintenance processes are impaired in DS [50, 51]. In contrast, lower MAST in GO is in agreement with the notion that offspring of long-lived parents show slower ageing [43, 52] and improved telomere length maintenance [53–55] compared to normal ageing subjects.

To investigate possible sources of variation of MAST in the general population, we next adopted a multidimensional approach that integrates demographic, dietary/lifestyle and clinical data.

Concerning demographic variables, a variation of MAST was found by country. This association, which was predominantly driven by samples from Austria, seems to be spurious and generated by the effect of third variables (e.g. age, BMI, alcohol and white bread consumption; see Table S12).

Although nutritional and lifestyle factors are believed to be significant modifiers of epigenetic patterns, MAST was independent of most of them except for the consumption of white bread and alcoholic beverages.

The positive relationship between white bread consumption and MAST is difficult to interpret since there are no previous reports regarding this association. However, there is initial evidence that dietary patterns, typified by higher intakes of refined grains, are associated with DNA methylation alterations [56] and telomere shortening [57]. The possible underlying mechanisms include epigenetic alterations induced by higher oxidative stress or an imbalanced supply of nutrients that act as methyl donors or cofactors in the one-carbon metabolism pathway.

The association of MAST with alcohol consumption is in line with previous observations [58, 59] and further supports the idea that chronic alcohol consumption influences genomic DNA methylation pattern. Within subtelomeric DNA, alcohol-induced hypermethylation is likely to have an impact on the telomere length homeostasis. In fact, subtelomeric DNA hypermethylation has been associated with telomere shortening in ethanol-treated cells [60].

A multitude of studies indicate a relationship between telomere shortening in blood cells and dyslipidaemia- and hyperglycaemia-related diseases (reviewed in [61]). Furthermore, recent follow-up studies have advocated that telomere shortening is a long-term marker of cellular ageing, illustrating a chronic deterioration of a person’s metabolic health [62]. In this context, the positive association of MAST with total blood triglycerides and LDL cholesterol is likely to be a reflection of an underlying mechanism translating the increased cell damage and turnover that are secondary to elevated blood lipid levels, to shortened telomere length [62–64].

Similarly, the positive association of MAST with fibrinogen levels is in agreement with previous studies that report shorter telomeres in the blood cells of individuals with higher levels of fibrinogen, an association that probably reflects cardiovascular ageing [65, 66].

The positive association of MAST with DNMT1 and DNMT3A and its negative association with TET3 transcript levels suggest that the age-associated methylation increase of the two subtelomeric regions reflects in part expression changes of components of the DNA methylation/demethylation machinery [36, 67]. This possibility is in line with previous data showing that the modulation of the DNMT and TET expression has an impact on subtelomeric methylation *status* [6, 68–70].

Finally, the association of MAST with specific white blood cell counts may reflect pieces of evidence that white blood cell types have different rates of telomere length changes with age, which would imply differential age-related subtelomeric methylation changes in individual cell types. Specifically, the positive association of MAST with lymphocyte counts agrees with previous findings showing that this leucocyte subpopulation (especially the B-cells) exhibits higher rates of telomere loss with ageing compared to other components of the PBMC [71]. It cannot be ruled out, however, that associations between MAST and white blood cell counts are also influenced by the effect of ageing on lymphocyte subpopulation numbers. The age-related decrease of lymphocyte counts [71, 72] may be an additional mediating factor in the MAST–lymphocyte count association. The inverse association of MAST with neutrophils, which are not part of the PBMC fraction where MAST was measured, may reflect their decrease in numbers with ageing [72].

After the identification of factors associated with MAST variations in the general population, we then sought to determine their impact on the differences in MAST between age groups. The results showed that, although all the critical variables were taken into account in the analysis, the differences of MAST between the age groups were still significant (Supplementary Table S12). This would indicate that age predicts subtelomeric DNA methylation changes in PBMC as an almost independent variable with respect to all other variables and factors evaluated here. This result may be reminiscent of recent data showing that age-related changes of specific CpGs in blood, which are part of Horvath’s epigenetic ageing clock (Horvath’s pan tissue 353 CpG site clock [73]), primarily reflect cell-intrinsic properties [74, 75]. In contrast, the influence of “extrinsic” factors is weak, but it is nevertheless driven by multiple environmental and lifestyle factors [76]. Nevertheless, none of Horvath’s epigenetic clock CpGs is located in or near the subtelomeric regions here analysed. This points to the existence of different pathways/mechanisms underlying the clock and the epigenetic ageing of the subtelomeres, as suggested by the observation that the length of telomeres and the Horvath’s epigenetic clock are independently associated with chronological age and do not correlate with each other [77].

This analysis also confirmed alcohol consumption, the expression of DNMT1, blood levels of glycated haemoglobin and glucose as important explanatory factors. All other factors were not associated with MAST in the GLM or olny indirectly linked to it. For example, recruitment centre and sex seem to have an effect on MAST through other exogenous variables, including dietary/lifestyle factors (i.e. alcohol and white bread consumption), markers of carbohydrates (i.e. serum glucose level) and lipid metabolism (i.e. blood levels of LDL cholesterol, triglycerides and free fatty acids).

On the other hand, the differences in MAST between GO, SGO and RASIG seem to depend on multiple factors, ranging from dietary habits (i.e. French fries consumption) and metabolic fitness markers (i.e. BMI and blood levels of glycated haemoglobin and glucose) to molecular markers (i.e. DNMT1 expression).

These data suggest that if a more efficient control of the epigenetic *status* of the subtelomeres represents a further element of the advantages that predispose the GO to healthy ageing, this could be related to a peculiar physiology that confers to the offspring of long-lived subjects favourable body anthropometrical characteristics [78] and better glucose handling [79, 80].

Collectively, results from this study confirm in large-scale population settings that ageing has an impact on the methylation profile of subtelomeric DNA. These data, together with the identification of factors that are associated with subtelomeric DNA methylation variation, could be helpful for future research aimed at understanding the mechanisms underlying the ageing process.

## Supplementary information

ESM 1 (DOCX 15 kb)

ESM 2 (PDF 537 kb)

## Data Availability

The data that support the findings of this study are available from the authors upon reasonable request.

## References

[1] Linardopoulou EV, Williams EM, Fan Y, Friedman C, Young JM, Trask BJ (2005). Human subtelomeres are hot spots of interchromosomal recombination and segmental duplication. Nature..

[2] Mefford HC, Trask BJ (2002). The complex structure and dynamic evolution of human subtelomeres. Nature reviews..

[3] Riethman H, Ambrosini A, Paul S (2005). Human subtelomere structure and variation. Chromosom Res.

[4] Blasco MA (2007). The epigenetic regulation of mammalian telomeres. Nature reviews..

[5] Mikkelsen TS, Ku M, Jaffe DB, Issac B, Lieberman E, Giannoukos G, Alvarez P, Brockman W, Kim TK, Koche RP, Lee W, Mendenhall E, O’Donovan A, Presser A, Russ C, Xie X, Meissner A, Wernig M, Jaenisch R, Nusbaum C, Lander ES, Bernstein BE (2007). Genome-wide maps of chromatin state in pluripotent and lineage-committed cells. Nature..

[6] Gonzalo S, Jaco I, Fraga MF, Chen T, Li E, Esteller M, Blasco MA (2006). DNA methyltransferases control telomere length and telomere recombination in mammalian cells. Nat Cell Biol.

[7] Vera E, Canela A, Fraga MF, Esteller M, Blasco MA (2008). Epigenetic regulation of telomeres in human cancer. Oncogene..

[8] Feil R, Fraga MF (2012). Epigenetics and the environment: emerging patterns and implications. Nature reviews..

[9] Schubeler D (2015). Function and information content of DNA methylation. Nature..

[10] Lopez-Otin C, Blasco MA, Partridge L, Serrano M, Kroemer G (2013). The hallmarks of aging. Cell..

[11] Ciccarone F, Tagliatesta S, Caiafa P, Zampieri M (2018). DNA methylation dynamics in aging: how far are we from understanding the mechanisms?. Mech Ageing Dev.

[12] Pal S, Tyler JK (2016). Epigenetics and aging. Sci Adv.

[13] Bell CG, Lowe R, Adams PD, Baccarelli AA, Beck S, Bell JT, Christensen BC, Gladyshev VN, Heijmans BT, Horvath S, Ideker T, Issa JPJ, Kelsey KT, Marioni RE, Reik W, Relton CL, Schalkwyk LC, Teschendorff AE, Wagner W, Zhang K, Rakyan VK (2019). DNA methylation aging clocks: challenges and recommendations. Genome Biol.

[14] Horvath S, Raj K (2018). DNA methylation-based biomarkers and the epigenetic clock theory of ageing. Nature reviews.

[15] Zhang Q, Vallerga CL, Walker RM, Lin T, Henders AK, Montgomery GW, He J, Fan D, Fowdar J, Kennedy M, Pitcher T, Pearson J, Halliday G, Kwok JB, Hickie I, Lewis S, Anderson T, Silburn PA, Mellick GD, Harris SE, Redmond P, Murray AD, Porteous DJ, Haley CS, Evans KL, McIntosh AM, Yang J, Gratten J, Marioni RE, Wray NR, Deary IJ, McRae AF, Visscher PM (2019). Improved precision of epigenetic clock estimates across tissues and its implication for biological ageing. Genome medicine.

[16] Levine ME, Lu AT, Quach A, Chen BH, Assimes TL, Bandinelli S, Hou L, Baccarelli AA, Stewart JD, Li Y, Whitsel EA, Wilson JG, Reiner AP, Aviv A, Lohman K, Liu Y, Ferrucci L, Horvath S (2018). An epigenetic biomarker of aging for lifespan and healthspan. Aging..

[17] Hu H, Li B, Duan S (2019). The alteration of subtelomeric DNA methylation in aging-related diseases. Front Genet.

[18] Buxton JL, Suderman M, Pappas JJ, Borghol N, McArdle W, Blakemore AI (2014). Human leukocyte telomere length is associated with DNA methylation levels in multiple subtelomeric and imprinted loci. Sci Rep.

[19] Guan JZ, Guan WP, Maeda T, Makino N (2012). The subtelomere of short telomeres is hypermethylated in Alzheimer’s disease. Aging Dis.

[20] Guan JZ, Guan WP, Maeda T, Makino N (2013). Analysis of telomere length and subtelomeric methylation of circulating leukocytes in women with Alzheimer’s disease. Aging Clin Exp Res.

[21] Maeda T, Guan JZ, Koyanagi M, Higuchi Y, Makino N (2012). Aging-associated alteration of telomere length and subtelomeric status in female patients with Parkinson’s disease. J Neurogenet.

[22] Maeda T, Guan JZ, Oyama J, Higuchi Y, Makino N (2009). Aging-associated alteration of subtelomeric methylation in Parkinson’s disease. J Gerontol A Biol Sci Med Sci.

[23] Makino N, Maeda T, Abe N (2019). Short telomere subtelomeric hypomethylation is associated with telomere attrition in elderly diabetic patients. Can J Physiol Pharmacol.

[24] Choudhury SR, Cui Y, Milton JR, Li J, Irudayaraj J (2015). Selective increase in subtelomeric DNA methylation: an epigenetic biomarker for malignant glioma. Clin Epigenetics.

[25] Han Y, Xu J, Kim J, Wu X, Gu J (2017). Methylation of subtelomeric repeat D4Z4 in peripheral blood leukocytes is associated with biochemical recurrence in localized prostate cancer patients. Carcinogenesis..

[26] Lee ME, Rha SY, Jeung HC, Chung HC, Oh BK (2009). Subtelomeric DNA methylation and telomere length in human cancer cells. Cancer Lett.

[27] Oh BK, Um TH, Choi GH, Park YN (2011). Frequent changes in subtelomeric DNA methylation patterns and its relevance to telomere regulation during human hepatocarcinogenesis. Int J Cancer.

[28] Xu J, Tsai CW, Chang WS, Han Y, Bau DT, Pettaway CA, Gu J (2019). Methylation of global DNA repeat LINE-1 and subtelomeric DNA repeats D4Z4 in leukocytes is associated with biochemical recurrence in African American prostate cancer patients. Carcinogenesis..

[29] Maeda T, Guan JZ, Oyama J, Higuchi Y, Makino N (2009). Age-related changes in subtelomeric methylation in the normal Japanese population. J Gerontol A Biol Sci Med Sci.

[30] Burkle A, Moreno-Villanueva M, Bernhard J, Blasco M, Zondag G, Hoeijmakers JH (2015). MARK-AGE biomarkers of ageing. Mech Ageing Dev.

[31] Capri M, Moreno-Villanueva M, Cevenini E, Pini E, Scurti M, Borelli V, Palmas MG, Zoli M, Schön C, Siepelmeyer A, Bernhardt J, Fiegl S, Zondag G, de Craen AJM, Hervonen A, Hurme M, Sikora E, Gonos ES, Voutetakis K, Toussaint O, Debacq-Chainiaux F, Grubeck-Loebenstein B, Bürkle A, Franceschi C (2015). MARK-AGE population: from the human model to new insights. Mech Ageing Dev.

[32] Moreno-Villanueva M, Kotter T, Sindlinger T, Baur J, Oehlke S, Burkle A (2015). The MARK-AGE phenotypic database: structure and strategy. Mech Ageing Dev.

[33] Moreno-Villanueva M, Capri M, Breusing N, Siepelmeyer A, Sevini F, Ghezzo A, Craen AJM, Hervonen A, Hurme M, Schön C, Grune T, Franceschi C, Bürkle A (2015). MARK-AGE standard operating procedures (SOPs): a successful effort. Mech Ageing Dev.

[34] Baur J, Kotter T, Moreno-Villanueva M, Sindlinger T, Berthold MR, Burkle A (2015). The MARK-AGE extended database: data integration and pre-processing. Mech Ageing Dev.

[35] Baur J, Moreno-Villanueva M, Kotter T, Sindlinger T, Burkle A, Berthold MR (2015). MARK-AGE data management: cleaning, exploration and visualization of data. Mech Ageing Dev.

[36] Ciccarone F, Malavolta M, Calabrese R, Guastafierro T, Bacalini MG, Reale A, Franceschi C, Capri M, Hervonen A, Hurme M, Grubeck-Loebenstein B, Koller B, Bernhardt J, Schӧn C, Slagboom PE, Toussaint O, Sikora E, Gonos ES, Breusing N, Grune T, Jansen E, Dollé M, Moreno-Villanueva M, Sindlinger T, Bürkle A, Zampieri M, Caiafa P (2016). Age-dependent expression of DNMT1 and DNMT3B in PBMCs from a large European population enrolled in the MARK-AGE study. Aging Cell.

[37] Zampieri M, Ciccarone F, Palermo R, Cialfi S, Passananti C, Chiaretti S, Nocchia D, Talora C, Screpanti I, Caiafa P (2014). The epigenetic factor BORIS/CTCFL regulates the NOTCH3 gene expression in cancer cells. Biochim Biophys Acta.

[38] Suchiman HE, Slieker RC, Kremer D, Slagboom PE, Heijmans BT, Tobi EW (2015). Design, measurement and processing of region-specific DNA methylation assays: the mass spectrometry-based method EpiTYPER. Front Genet.

[39] Franceschi C, Bezrukov V, Blanche H, Bolund L, Christensen K, de Benedictis G (2007). Genetics of healthy aging in Europe: the EU-integrated project GEHA (GEnetics of Healthy Aging). Ann N Y Acad Sci.

[40] Ambrosini A, Paul S, Hu S, Riethman H (2007). Human subtelomeric duplicon structure and organization. Genome Biol.

[41] Young E, Abid HZ, Kwok PY, Riethman H, Xiao M (2020). Comprehensive analysis of human subtelomeres by whole genome mapping. PLoS Genet.

[42] Maeda T, Guan JZ, Higuchi Y, Oyama J, Makino N (2009). Aging-related alterations of subtelomeric methylation in sarcoidosis patients. J Gerontol A Biol Sci Med Sci.

[43] Atzmon G, Rincon M, Rabizadeh P, Barzilai N (2005). Biological evidence for inheritance of exceptional longevity. Mech Ageing Dev.

[44] Patterson D, Cabelof DC (2012). Down syndrome as a model of DNA polymerase beta haploinsufficiency and accelerated aging. Mech Ageing Dev.

[45] Yehezkel S, Rebibo-Sabbah A, Segev Y, Tzukerman M, Shaked R, Huber I, Gepstein L, Skorecki K, Selig S (2011). Reprogramming of telomeric regions during the generation of human induced pluripotent stem cells and subsequent differentiation into fibroblast-like derivatives. Epigenetics..

[46] Elliott G, Hong C, Xing X, Zhou X, Li D, Coarfa C, Bell RJA, Maire CL, Ligon KL, Sigaroudinia M, Gascard P, Tlsty TD, Harris RA, Schalkwyk LC, Bilenky M, Mill J, Farnham PJ, Kellis M, Marra MA, Milosavljevic A, Hirst M, Stormo GD, Wang T, Costello JF (2015). Intermediate DNA methylation is a conserved signature of genome regulation. Nat Commun.

[47] Slieker RC, van Iterson M, Luijk R, Beekman M, Zhernakova DV, Moed MH (2016). Age-related accrual of methylomic variability is linked to fundamental ageing mechanisms. Genome Biol.

[48] Robin JD, Ludlow AT, Batten K, Magdinier F, Stadler G, Wagner KR, Shay JW, Wright WE (2014). Telomere position effect: regulation of gene expression with progressive telomere shortening over long distances. Genes Dev.

[49] Chu HP, Cifuentes-Rojas C, Kesner B, Aeby E, Lee HG, Wei C, Oh HJ, Boukhali M, Haas W, Lee JT (2017). TERRA RNA antagonizes ATRX and protects telomeres. Cell..

[50] Brando B, Longo A, Beltrami B, Passoni D, Verna R, Licastro F, Corsi MM (2004). Determination of telomere length by flow-fluorescence in situ hybridization in Down’s syndrome patients. Int J Tissue React.

[51] Vaziri H, Schachter F, Uchida I, Wei L, Zhu X, Effros R (1993). Loss of telomeric DNA during aging of normal and trisomy 21 human lymphocytes. Am J Hum Genet.

[52] Horvath S, Pirazzini C, Bacalini MG, Gentilini D, Di Blasio AM, Delledonne M (2015). Decreased epigenetic age of PBMCs from Italian semi-supercentenarians and their offspring. Aging..

[53] Arai Y, Martin-Ruiz CM, Takayama M, Abe Y, Takebayashi T, Koyasu S, Suematsu M, Hirose N, von Zglinicki T (2015). Inflammation, but not telomere length, predicts successful ageing at extreme old age: a longitudinal study of semi-supercentenarians. EBioMedicine..

[54] Atzmon G, Cho M, Cawthon RM, Budagov T, Katz M, Yang X, Siegel G, Bergman A, Huffman DM, Schechter CB, Wright WE, Shay JW, Barzilai N, Govindaraju DR, Suh Y (2010). Evolution in health and medicine Sackler colloquium: genetic variation in human telomerase is associated with telomere length in Ashkenazi centenarians. Proc Natl Acad Sci U S A.

[55] Tedone E, Arosio B, Gussago C, Casati M, Ferri E, Ogliari G, Ronchetti F, Porta A, Massariello F, Nicolini P, Mari D (2014). Leukocyte telomere length and prevalence of age-related diseases in semisupercentenarians, centenarians and centenarians’ offspring. Exp Gerontol.

[56] Colacino JA, Arthur AE, Dolinoy DC, Sartor MA, Duffy SA, Chepeha DB, Bradford C, Walline H, Mchugh J, D’SILVA N, Carey T, Wolf G, Taylor J, Peterson K, Rozek LS (2012). Pretreatment dietary intake is associated with tumor suppressor DNA methylation in head and neck squamous cell carcinomas. Epigenetics..

[57] Garcia-Calzon S, Moleres A, Martinez-Gonzalez MA, Martinez JA, Zalba G, Marti A (2015). Dietary total antioxidant capacity is associated with leukocyte telomere length in a children and adolescent population. Clin Nutr.

[58] Liu C, Marioni RE, Hedman AK, Pfeiffer L, Tsai PC, Reynolds LM, Just AC, Duan Q, Boer CG, Tanaka T, Elks CE, Aslibekyan S, Brody JA, Kühnel B, Herder C, Almli LM, Zhi D, Wang Y, Huan T, Yao C, Mendelson MM, Joehanes R, Liang L, Love SA, Guan W, Shah S, McRae AF, Kretschmer A, Prokisch H, Strauch K, Peters A, Visscher PM, Wray NR, Guo X, Wiggins KL, Smith AK, Binder EB, Ressler KJ, Irvin MR, Absher DM, Hernandez D, Ferrucci L, Bandinelli S, Lohman K, Ding J, Trevisi L, Gustafsson S, Sandling JH, Stolk L, Uitterlinden AG, Yet I, Castillo-Fernandez JE, Spector TD, Schwartz JD, Vokonas P, Lind L, Li Y, Fornage M, Arnett DK, Wareham NJ, Sotoodehnia N, Ong KK, van Meurs JBJ, Conneely KN, Baccarelli AA, Deary IJ, Bell JT, North KE, Liu Y, Waldenberger M, London SJ, Ingelsson E, Levy D (2018). A DNA methylation biomarker of alcohol consumption. Mol Psychiatry.

[59] Wilson LE, Xu Z, Harlid S, White AJ, Troester MA, Sandler DP, Taylor JA (2019). Alcohol and DNA methylation: an epigenome-wide association study in blood and normal breast tissue. Am J Epidemiol.

[60] Harpaz T, Abumock H, Beery E, Edel Y, Lahav M, Rozovski U, et al. The effect of ethanol on telomere dynamics and regulation in human cells. Cells. 2018;7(10). 10.3390/cells7100169 PMC6210749 30326633

[61] Kirchner H, Shaheen F, Kalscheuer H, Schmid SM, Oster H, Lehnert H. The telomeric complex and metabolic disease. Genes (Basel). 2017;8(7). 10.3390/genes8070176 PMC5541309 28686177

[62] Revesz D, Milaneschi Y, Verhoeven JE, Penninx BW (2014). Telomere length as a marker of cellular aging is associated with prevalence and progression of metabolic syndrome. J Clin Endocrinol Metab.

[63] Harte AL, da Silva NF, Miller MA, Cappuccio FP, Kelly A, O’Hare JP (2012). Telomere length attrition, a marker of biological senescence, is inversely correlated with triglycerides and cholesterol in South Asian males with type 2 diabetes mellitus. Exp Diabetes Res.

[64] Weischer M, Bojesen SE, Cawthon RM, Freiberg JJ, Tybjaerg-Hansen A, Nordestgaard BG (2012). Short telomere length, myocardial infarction, ischemic heart disease, and early death. Arterioscler Thromb Vasc Biol.

[65] Masi S, Nightingale CM, Day IN, Guthrie P, Rumley A, Lowe GD (2012). Inflammation and not cardiovascular risk factors is associated with short leukocyte telomere length in 13- to 16-year-old adolescents. Arterioscler Thromb Vasc Biol.

[66] von Kanel R, Malan NT, Hamer M, van der Westhuizen FH, Malan L (2014). Leukocyte telomere length and hemostatic factors in a South African cohort: the SABPA Study. J Thromb Haemost.

[67] Valentini E, Zampieri M, Malavolta M, Bacalini MG, Calabrese R, Guastafierro T, Reale A, Franceschi C, Hervonen A, Koller B, Bernhardt J, Slagboom PE, Toussaint O, Sikora E, Gonos ES, Breusing N, Grune T, Jansen E, Dollé MET, Moreno-Villanueva M, Sindlinger T, Bürkle A, Ciccarone F, Caiafa P (2016). Analysis of the machinery and intermediates of the 5hmC-mediated DNA demethylation pathway in aging on samples from the MARK-AGE Study. Aging..

[68] El-Maarri O, Kareta MS, Mikeska T, Becker T, Diaz-Lacava A, Junen J (2009). A systematic search for DNA methyltransferase polymorphisms reveals a rare DNMT3L variant associated with subtelomeric hypomethylation. Hum Mol Genet.

[69] Yang J, Guo R, Wang H, Ye X, Zhou Z, Dan J, Wang H, Gong P, Deng W, Yin Y, Mao SQ, Wang L, Ding J, Li J, Keefe DL, Dawlaty MM, Wang J, Xu GL, Liu L (2016). Tet enzymes regulate telomere maintenance and chromosomal stability of mouse ESCs. Cell Rep.

[70] Yehezkel S, Segev Y, Viegas-Pequignot E, Skorecki K, Selig S (2008). Hypomethylation of subtelomeric regions in ICF syndrome is associated with abnormally short telomeres and enhanced transcription from telomeric regions. Hum Mol Genet.

[71] Lin Y, Damjanovic A, Metter EJ, Nguyen H, Truong T, Najarro K, Morris C, Longo DL, Zhan M, Ferrucci L, Hodes RJ, Weng NP (2015). Age-associated telomere attrition of lymphocytes in vivo is co-ordinated with changes in telomerase activity, composition of lymphocyte subsets and health conditions. Clin Sci (Lond).

[72] Valiathan R, Ashman M, Asthana D (2016). Effects of ageing on the immune system: infants to elderly. Scand J Immunol.

[73] Horvath S (2013). DNA methylation age of human tissues and cell types. Genome Biol.

[74] Matsuyama M, WuWong DJ, Horvath S, Matsuyama S (2019). Epigenetic clock analysis of human fibroblasts in vitro: effects of hypoxia, donor age, and expression of hTERT and SV40 largeT. Aging..

[75] Soraas A, Matsuyama M, de Lima M, Wald D, Buechner J, Gedde-Dahl T (2019). Epigenetic age is a cell-intrinsic property in transplanted human hematopoietic cells. Aging Cell.

[76] Quach A, Levine ME, Tanaka T, Lu AT, Chen BH, Ferrucci L, Ritz B, Bandinelli S, Neuhouser ML, Beasley JM, Snetselaar L, Wallace RB, Tsao PS, Absher D, Assimes TL, Stewart JD, Li Y, Hou L, Baccarelli AA, Whitsel EA, Horvath S (2017). Epigenetic clock analysis of diet, exercise, education, and lifestyle factors. Aging..

[77] Marioni RE, Harris SE, Shah S, McRae AF, von Zglinicki T, Martin-Ruiz C, Wray NR, Visscher PM, Deary IJ (2018). The epigenetic clock and telomere length are independently associated with chronological age and mortality. Int J Epidemiol.

[78] Bucci L, Ostan R, Cevenini E, Pini E, Scurti M, Vitale G, Mari D, Caruso C, Sansoni P, Fanelli F, Pasquali R, Gueresi P, Franceschi C, Monti D (2016). Centenarians’ offspring as a model of healthy aging: a reappraisal of the data on Italian subjects and a comprehensive overview. Aging..

[79] Rozing MP, Westendorp RG, de Craen AJ, Frolich M, de Goeij MC, Heijmans BT (2010). Favorable glucose tolerance and lower prevalence of metabolic syndrome in offspring without diabetes mellitus of nonagenarian siblings: the Leiden longevity study. J Am Geriatr Soc.

[80] Wijsman CA, Rozing MP, Streefland TC, le Cessie S, Mooijaart SP, Slagboom PE (2011). Familial longevity is marked by enhanced insulin sensitivity. Aging Cell.

