# Supplementary material for: Ageing affects subtelomeric DNA methylation in blood cells from a large European population enrolled in the MARK-AGE study
Source: GeroScience. 2021 Apr 19;43(3):1283–302. doi: 10.1007/s11357-021-00347-9 (PMC8190237; doi:10.1007/s11357-021-00347-9)
Supplement: Supplementary file 1 — (DOCX 15 kb) [file 11357_2021_347_MOESM1_ESM.docx]

**Supplementary list of abbreviations and their definition**

**(Abbreviation: definition)**

BMI: body mass index defined as the body mass (kg) divided by the square of the body height (m).

C-reactive protein: concentration of C-reactive Protein in peripheral blood plasma.

Count of white blood cells, monocytes, lymphocytes, neutrophil, eosinophil and basophil: number of cells in peripheral blood.

Fibrinogen: concentration of Fibrinogen in peripheral blood plasma.

Free fatty acids: concentration of free fatty acids in peripheral blood serum.

Glucose: glucose concentration in peripheral blood serum.

Glycosylated haemoglobin A1C: percentage of glycosylated Haemoglobin A1C in peripheral blood.

High density lipoproteins cholesterol: peripheral blood serum concentration of cholesterol in HDL particles.

Homocysteine: concentration of Homocysteine in peripheral blood plasma.

Low density lipoproteins cholesterol: peripheral blood serum concentration of cholesterol in LDL particles.

Ratio of lymphocytes to monocytes: count of lymphocytes divided by the count of monocytes in peripheral blood.

Relative expression of DNMT1, -3A and 3B: transcript level of DNA Methyltransferase 1, 3A and 3B divided by the transcript level of Beta-Glucuronidase in PBMC.

Relative expression of TDG: transcript level of Thymine DNA Glycosylase divided by the transcript level of Beta-Glucuronidase in PBMC.

Relative expression of TET1, 2 and 3: transcript level of Ten-eleven translocation Methylcytosine Dioxygenase 1, 2 and 3 divided by the transcript level of Beta-Glucuronidase in PBMC.

Total cholesterol: total cholesterol concentration in peripheral blood serum.

Triglycerides: total triglycerides concentration in peripheral blood serum.
