# Supplementary material for: Ageing affects subtelomeric DNA methylation in blood cells from a large European population enrolled in the MARK-AGE study
Source: GeroScience. 2021 Apr 19;43(3):1283–302. doi: 10.1007/s11357-021-00347-9 (PMC8190237; doi:10.1007/s11357-021-00347-9)
Supplement: Supplementary file 2 — (PDF 537 kb) [file 11357_2021_347_MOESM2_ESM.pdf]

Table S1

Subtelomere		Age group	35	45	55	65	*P* KW*	*P* GLM[†]
		**Stat**	**a**	**b**	**c**	**d**		
**5p**	**CpG 1**	**Median**	0.680 (0.630-0.740) [b,c,d]	0.700 (0.640-0.760) [a,d]	0.715 (0.660-0.760) [a]	0.720 (0.660-0.775) [a,b]	**< 0.001**	**< 0.001**
		N	467	543	572	548		
	**CpG 2**	**Median**	0.760 (0.695-0.810) [d]	0.760 (0.700-0.810) [d]	0.760 (0.710-0.830)	0.780 (0.710-0.830) [a,b]	**0.014**	**0.037**
		N	460	521	575	540		
	**CpG 5**	**Median**	0.640 (0.560-0.700) [c,d]	0.650 (0.580-0.710)	0.650 (0.590-0.730) [a]	0.660 (0.580-0.750) [a]	**< 0.001**	**< 0.001**
		N	469	532	583	557		
	**5p mean**	**Median**	0.763 (0.735-0.795) [c,d]	0.773 (0.738-0.805) [d]	0.778 (0.743-0.810) [a]	0.788 (0.750-0.818) [a,b]	**< 0.001**	**< 0.001**
		N	468	537	579	552		
**21q**	**CpG 1.2**	**Median**	0.430 (0.360-0.520)	0.430 (0.370-0.510)	0.440 (0.360-0.520)	0.450 (0.370-0.540)	0.660	**0.018**
		N	469	533	578	551		
	**CpG 3**	**Median**	0.480 (0.410-0.550) [d]	0.480 (0.420-0.560) [d]	0.480 (0.420-0.560)	0.500 (0.420-0.590) [a,b]	**0.002**	**< 0.001**
		N	466	535	572	553		
	**CpG 4**	**Median**	0.640 (0.560-0.730)	0.650 (0.580-0.720)	0.650 (0.570-0.730)	0.660 (0.570-0.755)	**0.047**	**0.009**
		N	468	538	579	560		
	**CpG 5**	**Median**	0.390 (0.340-0.460) [d]	0.400 (0.350-0.450)	0.400 (0.340-0.460)	0.410 (0.350-0.480) [a]	**0.016**	**0.002**
		N	467	535	577	554		
	**CpG 8**	**Median**	0.560 (0.490-0.620) [d]	0.560 (0.500-0.630) [d]	0.570 (0.500-0.640) [d]	0.590 (0.510-0.670) [a,b,c]	**< 0.001**	**< 0.001**
		N	465	534	579	556		
	**CpG 10-14**	**Median**	0.650 (0.580-0.710) [d]	0.650 (0.590-0.720) [d]	0.660 (0.590-0.730) [d]	0.670 (0.600-0.750) [a,b,c]	**< 0.001**	**< 0.001**
		N	468	540	581	559		
	**CpG 15**	**Median**	0.700 (0.640-0.760) [d]	0.710 (0.650-0.770) [d]	0.710 (0.650-0.780)	0.730 (0.660-0.800) [a,b]	**< 0.001**	**< 0.001**
		N	469	540	583	558		
	**CpG 16.17**	**Median**	0.580 (0.510-0.660) [d]	0.580 (0.520-0.655) [d]	0.590 (0.520-0.670) [d]	0.610 (0.540-0.700) [a,b,c]	**< 0.001**	**< 0.001**
		N	472	540	584	554		
	**CpG 18.19**	**Median**	0.640 (0.580-0.700) [c,d]	0.650 (0.590-0.720) [d]	0.660 (0.600-0.730) [a,d]	0.670 (0.610-0.760) [a,b,c]	**< 0.001**	**< 0.001**
		N	466	541	581	558		
	**CpG 20.21**	**Median**	0.715 (0.660-0.770) [c,d]	0.720 (0.670-0.790) [d]	0.730 (0.670-0.800) [a]	0.750 (0.680-0.820) [a,b]	**< 0.001**	**< 0.001**
		N	466	537	586	556		
	**21q mean**	**Median**	0.616 (0.553-0.679) [d]	0.623 (0.569-0.690) [d]	0.630 (0.564-0.701) [d]	0.649 (0.575-0.724) [a,b,c]	**< 0.001**	**< 0.001**
		N	473	545	589	560		

Table S2

			Parametric*	Non parametric*
**5p**	**CpG 1**	Correlation coefficient	0.149	0.155
		Sig. (2-tailed)	**< 0.001**	**< 0.001**
		95% C.I.	0.108; 0.193	0.115; 0.199
		N	2130	2130
	**CpG 2**	Correlation coefficient	0.052	0.068
		Sig. (2-tailed)	**0.018**	**0.002**
		95% C.I.	0.010; 0.096	0.027; 0.110
		N	2096	2096
	**CpG 5**	Correlation coefficient	0.100	0.098
		Sig. (2-tailed)	**< 0.001**	**< 0.001**
		95% C.I.	0.057; 0.142	0.053; 0.144
		N	2141	2141
	**Mean**	Correlation coefficient	0.132	0.140
		Sig. (2-tailed)	**< 0.001**	**< 0.001**
		95% C.I.	0.090; 0.173	0.100; 0.192
		N	2136	2136
**21q**	**CpG 1.2**	Correlation coefficient	0.063	0.057
		Sig. (2-tailed)	**0.003**	**0.008**
		95% C.I.	0.025; 0.105	0.018; 0.099
		N	2131	2131
	**CpG 3**	Correlation coefficient	0.087	0.080
		Sig. (2-tailed)	**< 0.001**	**< 0.001**
		95% C.I.	0.042; 0.126	0.035; 0.121
		N	2126	2126
	**CpG 4**	Correlation coefficient	0.059	0.049
		Sig. (2-tailed)	**0.006**	**0.022**
		95% C.I.	0.017; 0.102	0.007; 0.095
		N	2145	2145
	**CpG 5**	Correlation coefficient	0.072	0.064
		Sig. (2-tailed)	**0.001**	**0.003**
		95% C.I.	0.030; 0.116	0.021; 0.104
		N	2133	2133
	**CpG 8**	Correlation coefficient	0.108	0.096
		Sig. (2-tailed)	**< 0.001**	**< 0.001**
		95% C.I.	0.062; 0.149	0.053; 0.138
		N	2134	2134
	**10.11.12.1**	Correlation coefficient	0.096	0.091
		Sig. (2-tailed)	**< 0.001**	**< 0.001**
		95% C.I.	0.055; 0.134	0.048; 0.135
		N	2148	2148
	**CpG 15**	Correlation coefficient	0.101	0.099
		Sig. (2-tailed)	**< 0.001**	**< 0.001**
		95% C.I.	0.056; 0.141	0.060; 0.142
		N	2150	2150
	**CpG 16.17**	Correlation coefficient	0.103	0.098
		Sig. (2-tailed)	**< 0.001**	**< 0.001**
		95% C.I.	0.062; 0.143	0.057; 0.140
		N	2150	2150
	**CpG 18.19**	Correlation coefficient	0.132	0.128
		Sig. (2-tailed)	**< 0.001**	**< 0.001**
		95% C.I.	0.090; 0.174	0.085; 0.166
		N	2146	2146
	**CpG 20.21**	Correlation coefficient	0.118	0.114
		Sig. (2-tailed)	**< 0.001**	**< 0.001**
		95% C.I.	0.076; 0.160	0.072; 0.155
		N	2145	2145
	**Mean**	Correlation coefficient	0.111	0.105
		Sig. (2-tailed)	**< 0.001**	**< 0.001**
		95% C.I.	0.065; 0.156	0.063; 0.148
		N	2146	2146

Table S3

	Method	Method details	R vs Age (Pearson)	p	R vs Age (Spearman)	p
1	Linear Regression	All variables entered in a single step (Enter)	0.176	**< 0.001**	0.174	**< 0.001**
2	Linear Regression	Stepwise	0.162	**< 0.001**	0.160	**< 0.001**
3	Linear Regression	Backward	0.167	**< 0.001**	0.163	**< 0.001**
4	Automatic Linear regression	Standard Model (Forward method)	0.153	**< 0.001**	0.155	**< 0.001**
5	Automatic Linear regression	Standard Model (Enter method)	0.158	**<0.001**	0.156	**< 0.001**
6	Automatic Linear regression	Boosting (Enter method)	0.156	**< 0.001**	0.162	**< 0.001**
7	Linear Regression (after log transformation)	All variables entered in a single step (Enter)	0.172	**< 0.001**	0.171	**< 0.001**
8	Linear regression with variables selected by Principal Component Analysis	Factor Analysis	0.133	**< 0.001**	0.132	**< 0.001**

Table S4

Linear regression model*

R	R^2	Adjusted R^2	Estimate S.E.	F (anova)	Sig. (anova)
0.254	0.065	0.058	11.009	10.330	< 0.001

**Coefficients**		**USTD**		**STD**			**Bootstrap for Coefficients**			**95% C.I.**	
		**B**	**S.E.**	**Beta**	**t**	**Sig.**	**Bias**	**S.E.**	**Sig.**	**Lower**	**Upper**
	**(Constant)**	21.206	4.516		4.696	**< 0.001**	0.076	4.413	**0.001**	12.427	29.883
**5p**	**CpG 1**	17.567	3.278	0.124	5.360	**< 0.001**	0.012	3.224	**0.001**	11.500	23.882
	**CpG 2**	2.043	3.009	0.016	0.679	0.497	0.016	3.121	0.520	-3.603	8.159
	**CpG 5**	7.568	2.485	0.069	3.045	**0.002**	-0.031	2.504	**0.001**	2.641	12.413
**21q**	**CpG 1.2**	-1.100	3.830	-0.011	-0.287	0.774	-0.116	3.717	0.757	-8.739	5.916
	**CpG 3**	6.686	6.997	0.062	0.956	0.339	0.081	6.680	0.315	-6.970	20.291
	**CpG 4**	-23.427	5.246	-0.235	-4.466	**< 0.001**	0.133	5.047	**0.001**	-32.629	-13.344
	**CpG 5**	-1.450	7.021	-0.011	-0.207	0.836	-0.134	7.093	0.834	-14.246	11.993
	**CpG 8**	-2.368	11.177	-0.022	-0.212	0.832	-0.242	11.029	0.818	-23.953	18.021
	**CpG 10-14**	-19.228	10.812	-0.162	-1.778	0.075	-0.560	11.052	0.083	-39.824	0.334
	**CpG 15**	10.848	9.198	0.084	1.179	0.238	-0.111	9.173	0.250	-6.926	28.140
	**CpG 16.17**	-5.261	12.636	-0.048	-0.416	0.677	0.380	11.870	0.689	-28.755	18.476
	**CpG 18.19**	35.614	11.300	0.292	3.152	**0.002**	0.519	11.277	**0.004**	13.066	58.387
	**CpG 20.21**	20.190	11.534	0.151	1.751	0.080	-0.121	11.262	0.073	-1.144	41.303

Table S5

	Stat	*N*	Median (IQ)	*P* KW[§]
**Age group, RASIG**				
**35**	a	494	54.828 (53.106 - 56.465) [b,c,d]	**<0.001**
**45**	b	576	55.215 (53.375 - 57.389) [a,c,d]	
**55**	c	628	56.106 (54.214 - 58.007) [a,b,d]	
**65**	d	610	56.688 (54.692 - 58.741) [a,b,c]	
**GO***	a	457	55.987 (54.188 - 57.736) [c]	**0.040**
**SGO***	b	242	56.244 (54.433 - 57.741)	
**RASIG***	c	1048	56.399 (54.421 - 58.295) [a]	
**DS†**	a	34	56.115 (54.611 - 58.080) [b]	**0.018**
**RASIG†**	b	197	54.977 (53.173 - 56.744) [a]	

Table S6

	MAST *vs* age		MAST *vs* telomere lenght		Telomere lenght *vs* age	
	**Spearman**	**Pearson**	**Spearman**	**Pearson**	**Spearman**	**Pearson**
**Correl. coeff.**	0.269	0.274	-0.049	-0.071	-0.151	-0.153
**Sig. (2-tailed)**	**< 0.001**	**< 0.001**	**0.050**	**0.005**	**< 0.001**	**< 0.001**
**95% C.I.**	0.219; 0.311	0.229; 0.317	-0.103; -0.004	-0.122; -0.019	-0.201; -0.102	-0.201; -0.105
**N**	1596		1596		1596	

Table S7

Factor	Stat	*N*	MAST<br>Median (IQ)	*P* KW* (ε^2)	*P* GLM† (η^2)
**Recruitment centre**					
Finland	**a**	93	56.159 (54.040 - 58.251) [c]	**<0.001** (0.016)	**<0.001** (0.015)[§]
Italy	**b**	398	55.650 (53.568 - 57.638)		
Austria	**c**	399	54.865 (52.980 - 57.068) [a,d,e,f,g]		
Greece	**d**	392	55.972 (54.238 - 57.958) [c]		
Poland	**e**	406	56.039 (54.052 - 57.895) [c]		
Belgium	**f**	262	55.971 (54.050 - 57.419) [c]		
Germany	**g**	358	55.838 (54.113 - 57.668) [c]		
**Sex**					
F	**a**	1189	55.865 (53.826 - 57.800)	0.153 (0.002)	0.054 (0.002)[‡]
M	**b**	1119	55.722 (53.761 - 57.555)		
**Classes of BMI (Kg/m^2)**					
< 25	**a**	1032	55.530 (53.577 - 57.378)	**0.002** (0.007)	0.085 (0.009)[ə]
25 to < 30	**b**	872	56.048 (54.131 - 57.866) [c]		
30 and over	**c**	399	55.845 (53.963 - 58.077) [b]		

Table S8

Factor		Stat	*N*	MAST Median (IQ)	*P* KW* (ε^2)	*P* GLM† (η^2)
**Vegetables consumption**	< 1 serv./day	a	902	55.702 (53.818 - 57.437)	0.378 (0.001)	0.884 (0.002)
	= 1 serv./day	b	1049	55.852 (53.831 - 57.855)		
	≥ 2 serv./day	c	357	55.722 (53.674 - 57.585)		
**Fruit consumption**	< 1 serv./day	a	810	55.600 (53.718 - 57.488)	0.083 (0.003)	0.975 (0.002)
	= 1 serv./day	b	926	55.852 (53.869 - 57.739)		
	≥ 2 serv./day	c	572	56.002 (53.712 - 57.834)		
**Meat consumption**	≤ 1 serv./week	a	183	55.726 (54.069 - 58.235)	0.381 (0.001)	0.7 (0.002)
	2-6 serv./week	b	1838	55.844 (53.825 - 57.698)		
	≥ 7 serv./week	c	287	55.484 (53.528 - 57.295)		
**Vitamin consumption**	No serv./day	a	1441	55.771 (53.719 - 57.636)	0.469 (0.001)	0.906 (0.003)
	< 1 serv./day	b	570	55.813 (53.831 - 57.723)		
	≥ 1 serv./day	c	296	55.918 (54.156 - 57.784)		
**Dairy products consupmtion**	< 1 serv./weekly or never	a	267	55.588 (53.992 - 57.537)	0.306 (0.001)	0.537 (0.003)
	= 1-6 serv./weekly	b	771	55.777 (53.665 - 57.458)		
	≥ 1 serv./day	c	1270	55.832 (53.847 - 57.800)		
**Eggs consumption**	< 1 serv./weekly or never	a	1036	55.835 (53.812 - 57.809)	0.504 (< 0.001)	0.22 (0.002)
	≥ 1 serv./weekly	b	1272	55.788 (53.775 - 57.606)		
**Fish consumption**	< 1 serv./weekly or never	a	1044	55.778 (53.807 - 57.819)	0.771 (< 0.001)	0.089 (0.002)
	≥ 1 serv./weekly	b	1256	55.828 (53.776 - 57.541)		
**French fries consumption**	never	a	617	56.077 (54.062 - 58.115) c,b	**0.002** (0.006)	0.08 (0.008)
	< 1 serv./week	b	1315	55.745 (53.765 - 57.535) a		
	≥ 1 serv./week	c	357	55.671 (53.470 - 57.181) a		
**Brown bread Consuption**	< 1 serv./weekly or never	a	1154	55.794 (53.933 - 57.636)	0.065 (0.003)	0.189 (0.007)
	= 1-6 serv./weekly	b	641	55.563 (53.652 - 57.560)		
	≥ 1 serv./day	c	513	56.107 (53.784 - 57.983)		
**White bread consumption**	< 1 serv./weekly or never	a	863	55.942 (54.023 - 57.896)	**0.002** (0.007)	**0.034** (0.010)
	= 1-6 serv./weekly	b	805	55.401 (53.599 - 57.400)[c]		
	≥ 1 serv./day	c	629	55.916 (54.039 - 57.785)[b]		
**Whole bread consumption**	< 1 serv./weekly or never	a	326	55.930 (53.736 - 58.064)	0.055 (0.003)	0.614 (0.005)
	= 1-6 serv./weekly	b	1575	55.863 (53.865 - 57.770)		
	≥ 1 serv./day	c	405	55.365 (53.637 - 57.057)		
**Alchool consumption**	never	a	917	55.736 (53.761 - 57.776)	**0.023** (0.004)	**0.001** (0.006)
	Moderate consumption (max 7 serv /week)	b	816	55.615 (53.726 - 57.437)[c]		
	> 7 serv /week)	c	560	56.050 (54.198 - 57.819)[b]		
**Smoke habits**	never	a	1170	55.850 (53.767 - 57.674)	0.364 (0.001)	0.936 (0.004)
	former	b	708	55.835 (53.896 - 57.775)		
	current	c	430	55.605 (53.613 - 57.535)		

Table S9

Variable		Stat	*N*	MAST Median (IQ)	*P* KW* (ε^2)	*P* GLM† (η^2)
**Glucose (mmol/L) quartiles**	≤ 4.65	a	443	55.671 (53.764 - 57.310) [d]	**<0.001** (0.014)	0.062 (0.020)
	4.66 - 5.09	b	436	55.433 (53.391 - 57.277) [d]		
	5.10 - 5.57	c	418	55.953 (53.881 - 57.805)		
	≥ 5.58	d	356	56.324 (54.227 - 58.592) [a,b]		
**Glycosylated haemoglobin A1C (%) quartiles**	≤ 5.64	a	479	55.996 (54.181 - 57.512)	0.062 (0.005)	**< 0.001** (0.006)
	5.65 - 5.95	b	451	56.070 (53.902 - 58.030)		
	5.96 - 6.29	c	426	55.348 (53.358 - 57.406)		
	≥ 6.30	d	398	55.732 (53.826 - 57.809)		
**Total cholesterol (mg/dL) tertiles**	≤ 220	a	515	55.622 (53.576 - 57.190)	**0.009** (0.006)	0.080 (0.009)
	221-257	b	547	55.662 (53.764 - 57.730)		
	≥ 258	c	561	56.054 (54.137 - 58.019)		
**Triglycerides (mmol/L) quartiles**	< 0.78	a	429	55.353 (53.514 - 57.316) [d,b]	**<0.001** (0.013)	**0.001** (0.018)
	0.78 - 1.05	b	398	55.690 (53.625 - 57.730) [a]		
	1.06 - 1.50	c	401	55.805 (53.955 - 57.404) [d]		
	≥ 1.51	d	425	56.200 (54.275 - 58.287) [a,c]		
**High density lipoprotein cholesterol (mmol/L) quartiles**	≤ 1.20	a	428	55.771 (53.932 - 57.491)	0.252 (0.003)	0.112 (0.007)
	1.21 - 1.47	b	402	56.012 (53.933 - 58.101)		
	1.48 - 1.80	c	411	55.828 (53.847 - 57.776)		
	> 1.80	d	411	55.591 (53.576 - 57.629)		
**Low density lipoprotein cholesterol (mmol/L) quartiles**	≤ 2.7	a	416	55.707 (53.625 - 57.672)	**0.008** (0.008)	**0.038** (0.011)
	2.71 - 3.29	b	421	55.207 (53.591 - 57.500)		
	3.30 - 3.84	c	410	55.775 (53.859 - 57.536)		
	≥ 3.86	d	406	56.168 (54.254 - 58.137)		
**Free fatty acids (mg/dL) quartiles**	< 0.45	a	420	55.649 (53.608 - 57.246) [d]	**0.041** (0.006)	0.357 (0.009)
	0.45 - 0.60	b	411	55.814 (53.745 - 58.038)		
	0.61 - 0.79	c	420	55.622 (53.684 - 57.566)		
	≥ 0.80	d	400	56.097 (54.213 - 58.223) [a]		
**Homocysteine (µmol/L) quartiles**	≤ 11.05	a	464	55.840 (53.992 - 57.668)	0.605 (0.001)	0.078 (0.003)
	11.06 - 14.22	b	430	55.955 (53.875 - 57.667)		
	14.23 - 18.27	c	437	55.892 (53.793 - 57.842)		
	> 18.27	d	437	55.679 (53.822 - 57.595)		
**Fibrinogen (mg/ml) quartiles**	<2.538	a	547	54.994 (53.304 - 56.971) [b,c,d]	**<0.001** (0.024)	**0.008** (0.026)
	2.538-3.279	b	550	55.738 (53.672 - 57.599) [a,d]		
	3.280-4.197	c	554	56.086 (53.994 - 57.896) [a]		
	>4.197	d	548	56.195 (54.428 - 58.137) [a,b]		
**C-reactive protein (mg/l) quartiles**	<0.57	a	577	56.220 (54.317 - 57.684)	0.110 (0.008)	0.272 (0.010)
	0.57-1.17	b	576	56.378 (54.397 - 58.094)		
	1.18-2.42	c	571	56.682 (54.936 - 59.002)		
	>2.42	d	576	56.495 (54.463 - 58.388)		

Table S10

Variable		Stat	MAST *N*	Median (IQ)	*P* KW* (ε^2)	*P* GLM† (η^2)
**Relative DNMT1 expression, quartiles**	0.046-0.0980	a	460	55.826 (53.655 - 57.679) [d]	**0.001** (0.010)	**0.001** (0.015)
	0.0980-0.1293	b	418	55.416 (53.77 - 57.197) [d]		
	0.1294-0.1704	c	465	55.735 (53.712 - 57.666) [d]		
	>0.1704	d	420	56.363 (54.433 - 58.16) [a,b,c]		
**Relative DNMT3B expression, quartiles**	0.014-0.155	a	446	56.018 (54.042 - 57.821)	0.181 (0.003)	0.754 (0.004)
	0.156-0.205	b	413	56.002 (53.764 - 57.732)		
	0.206-0.281	c	450	55.967 (53.843 - 57.708)		
	>0.282	d	452	55.335 (53.866 - 57.448)		
**Relative DNMT3A expression, tertiles**	< 2.93	a	49	53.214 (51.367 - 54.866) [c]	**0.017** (0.228)	**0.001** (0.291)
	2.93-4.75	b	54	54.195 (53.147 - 56.331)		
	> 4.75	c	50	56.181 (54.169 - 58.329) [a]		
**Relative TET1 expression, tertiles**	0.047-0.149	a	49	55.623 (53.028 - 56.907)	0.529 (0.009)	0.134 (0.038)
	0.149-0.209	b	54	55.687 (54.417 - 57.398)		
	>0.209	c	50	55.548 (53.117 - 57.897)		
**Relative TET2 expression, tertiles**	0.616-3.604	a	53	56.312 (53.497 - 57.486)	0.282 (0.016)	0.524 (0.036)
	3.605-5.855	b	50	55.358 (53.029 - 57.398)		
	>5.855	c	50	55.364 (53.210 - 57.505)		
**Relative TET3 expression, tertiles**	1.366-2.638	a	51	56.166 (53.966 - 57.888) [c]	**0.027** (0.043)	**0.024** (0.068)
	2.639-3.363	b	54	55.971 (53.400 - 57.323)		
	>3.363	c	48	54.449 (52.590 - 56.808) [a]		
**Relative TDG expression, tertiles**	0.095-0.158	a	48	55.971 (53.764 - 57.78)	0.583 (0.007)	0.837 (0.014)
	0.159-0.209	b	42	55.364 (53.400 - 56.933)		
	>0.209	c	47	54.954 (53.029 - 57.486)		

Table S11

Variable		Stat	N	MAST Median (IQ)	P KW* (ε^2)	P GLM† (η^2)
**White blood cell count (n°x10^9/l)**	< 5.00	a	613	55.585 (53.655 - 57.636)	0.288 (0.002)	0.621 (0.003)
	5.00 - 5. 89	b	542	55.851 (53.978 - 57.601)		
	5.90 - 6.91	c	551	55.973 (53.955 - 57.805)		
	> 6.91	d	582	55.707 (53.769 - 57.639)		
**Monocyte count (n°/µl)**	< 364	a	477	55.624 (53.568 - 57.666)	0.322 (0.002)	0.645 (0.002)
	364 - 452	b	505	55.890 (53.909 - 57.658)		
	452 - 566	c	758	55.682 (53.736 - 57.668)		
	> 566	d	515	55.916 (54.113 - 57.8)		
**Lymphocyte count (n°/µl)**	< 1540	a	530	55.146 (53.381 - 57.303) [b,d]	**0.001** (0.011)	**<0.001** (0.008)
	1540 - 1890	b	499	55.994 (53.739 - 57.647) [a]		
	1891 - 2281	c	759	55.662 (53.794 - 57.732)		
	> 2281	d	468	56.120 (54.375 - 57.946) [a]		
**Lymphocytes to monocytes ratio**	< 3.35	a	522	55.415 (53.518 - 57.297) [d]	**0.035** (0.004)	**0.001** (0.009)
	3.35-4.20	b	520	55.687 (53.947 - 57.599)		
	4.20-5.24	c	773	55.798 (53.763 - 57.742)		
	> 5.24	d	441	56.033 (54.259 - 58.007) [a]		
**Neutrophil count (n°x10^9/l)**	< 2.56	a	480	56.104 (53.992 - 58.053) [d]	**0.024** (0.005)	**0.026** (0.009)
	2.56-3.20	b	508	55.616 (53.736 - 57.415)		
	3.20-3.90	c	775	55.808 (53.902 - 57.729)		
	≥ 3.99	d	500	55.481 (53.503 - 57.378) [a]		
**Eosinophil count (n°/µl)**	≥ 100	a	523	55.755 (53.774 - 57.464)	0.507 (0.001)	0.758 (0.003)
	101-151	b	502	55.892 (53.707 - 57.895)		
	152-230	c	776	55.616 (53.670 - 57.666)		
	≥ 231	d	460	55.885 (54.210 - 57.453)		
**Basophil count (n°/µl)**	≤ 20.4	a	531	55.784 (53.827 - 57.325)	0.636 (0.001)	0.882 (0.002)
	20.5-32.4	b	590	55.839 (53.950 - 57.895)		
	32.5-48.8	c	562	55.622 (53.585 - 57.729)		
	> 48.8	d	573	55.824 (53.833 - 57.611)		

Table S12

**Model**	**1**	**2**	**3**	**4**	**5**	**6**	**7**	**8**	**9**	**10**	**11**	**12**	**13**	**14**	**15**	**16**	**17**	**18**	**19**	**20**	**21**
**N**	1961	1961	1961	1916	1916	1916	1914	1913	1908	1801	1800	1552	1447	1915	1901	1900	1801	1801	1789	1758	1434
	**Wald**	**Wald**	**Wald**	**Wald**	**Wald**	**Wald**	**Wald**	**Wald**	**Wald**	**Wald**	**Wald**	**Wald**	**Wald**	**Wald**	**Wald**	**Wald**	**Wald**	**Wald**	**Wald**	**Wald**	**Wald**
	**Sig.**	**Sig.**	**Sig.**	**Sig.**	**Sig.**	**Sig.**	**Sig.**	**Sig.**	**Sig.**	**Sig.**	**Sig.**	**Sig.**	**Sig.**	**Sig.**	**Sig.**	**Sig.**	**Sig.**	**Sig.**	**Sig.**	**Sig.**	**Sig.**
**Age group**	134.571	133.299	134.080	130.890	131.350	131.054	133.570	111.090	127.429	116.492	117.190	101.278	72.680	116.452	119.128	131.372	115.957	105.258	115.782	81.760	64.037
	**< 0.001**	**< 0.001**	**< 0.001**	**< 0.001**	**< 0.001**	**< 0.001**	**< 0.001**	**< 0.001**	**< 0.001**	**< 0.001**	**< 0.001**	**< 0.001**	**< 0.001**	**< 0.001**	**< 0.001**	**< 0.001**	**< 0.001**	**< 0.001**	**< 0.001**	**< 0.001**	**< 0.001**
**Recruitment center**		2.203	2.181	3.533	3.503	3.509	4.205	3.426	4.146	3.598	3.094	2.764	2.635	4.426	3.347	3.107	2.989	3.763	3.868	5.008	3.647
		0.138	0.140	0.060	0.061	0.061	**0.040**	0.064	**0.042**	0.074	0.079	0.096	0.105	**0.035**	0.067	0.078	0.084	0.052	**0.049**	**0.025**	0.056
**Sex**			3.773	3.348	3.275	3.265	1.373	2.009	3.662	4.866	5.616	1.058	0.935	4.461	2.270	3.564	3.800	5.691	4.200	4.074	2.250
			0.052	0.067	0.070	0.071	0.241	0.156	0.056	**0.027**	**0.018**	0.604	0.334	**0.035**	0.132	0.059	0.051	**0.017**	**0.040**	**0.044**	0.134
**Lymphocytes to monocytes ratio**				3.310	3.371	3.334	3.345	3.289	3.194	2.839	2.525	2.353	1.779	3.720	2.690	3.309	2.731	2.572	3.126	2.554	1.935
				0.069	0.066	0.068	0.067	0.070	0.074	0.092	0.112	0.125	0.182	0.054	0.101	0.069	0.098	0.109	0.077	0.110	0.164
**Neuthophils**					0.644								0.552							0.453	0.426
					0.422								0.450							0.501	0.514
**Lymphocytes**						0.271							0.201							0.101	0.118
						0.603							0.654							0.751	0.731
**Alchool consumption**							6.426						4.011							4.952	3.820
							**0.011**						**0.045**							**0.026**	**0.050**
**Fibrinogen**								6.457					3.604							2.517	2.296
								**0.011**					0.058							0.113	0.130
**White bread consumption**									0.724				0.032							0.337	0.187
									0.395				0.858							0.562	0.666
**LDL-Cholesterol**										4.864			1.850							1.747	0.630
										**0.027**			0.174							0.186	0.427
**Triglycerides**											2.899		1.825							0.178	0.514
											0.089		0.177							0.673	0.474
**Relative DNMT1 expression**												7.481	4.578								4.398
												**0.006**	**0.032**								**0.036**
**BMI**														5.978						1.343	0.793
														**0.014**						0.247	0.373
**French fries consumption**															2.841					1.349	1.210
															0.092					0.245	0.271
**Glycated haemoglobin (HbA1c)**																1.576				9.273	9.015
																0.209				**0.002**	**0.003**
**Cholesterol**																	2.208			0.325	0.028
																	0.137			0.569	0.868
**Glucose**																		5.014		7.886	7.609
																		**0.025**		**0.005**	**0.006**
**Free fatty acids**																			1.638	0.874	0.287
																			0.201	0.350	0.592

Table S13

**Model**	**1**	**2**	**3**	**4**	**5**	**6**	**7**	**8**	**9**	**10**	**11**	**12**	**13**	**14**	**15**	**16**	**17**	**18**	**19**	**20**	**21**	**22**
**N**	1408	1408	1408	1408	1361	1361	1361	1359	1358	1358	1307	1307	1118	1069	1360	1350	1347	1307	1307	1304	1273	1057
	**Wald**	**Wald**	**Wald**	**Wald**	**Wald**	**Wald**	**Wald**	**Wald**	**Wald**	**Wald**	**Wald**	**Wald**	**Wald**	**Wald**	**Wald**	**Wald**	**Wald**	**Wald**	**Wald**	**Wald**	**Wald**	**Wald**
	**Sig.**	**Sig.**	**Sig.**	**Sig.**	**Sig.**	**Sig.**	**Sig.**	**Sig.**	**Sig.**	**Sig.**	**Sig.**	**Sig.**	**Sig.**	**Sig.**	**Sig.**	**Sig.**	**Sig.**	**Sig.**	**Sig.**	**Sig.**	**Sig.**	**Sig.**
**Subject group (GO, SGO, RASIG)**	12.935	13.170	13.503	12.483	12.504	12.511	12.504	11.126	11.842	13.598	10.497	9.892	10.960	8.485	10.894	11.247	12.013	10.420	9.627	10.350	6.224	5.480
	**0.002**	**0.001**	**0.001**	**0.002**	**0.002**	**0.002**	**0.002**	**0.004**	**0.003**	**0.001**	**0.005**	**0.007**	**0.004**	**0.014**	**0.004**	**0.004**	**0.002**	**0.005**	**0.008**	**0.006**	**0.045**	0.065
**Recruitment centre**		1.330	1.219	1.495	0.675	0.673	0.672	0.427	0.521	0.660	0.283	0.295	0.028	0.153	0.304	0.461	0.080	0.279	0.064	0.245	1.987	5.047
		0.249	0.270	0.222	0.411	0.412	0.412	0.513	0.470	0.417	0.595	0.587	0.867	0.695	0.581	0.497	0.777	0.598	0.786	0.621	0.159	**0.025**
**Sex**			1.441	1.810	1.673	1.681	1.679	1.875	0.933	1.207	1.789	2.121	0.585	0.518	2.079	1.731	0.866	1.505	2.448	2.057	2.045	1.238
			0.230	0.178	0.196	0.195	0.195	0.171	0.334	0.272	0.181	0.145	0.444	0.472	0.149	0.188	0.352	0.220	0.118	0.152	0.153	0.266
**Age**				16.330	15.993	15.974	15.968	16.303	15.533	15.842	15.947	14.917	13.841	12.077	15.652	17.048	13.121	15.265	13.623	14.737	12.592	11.409
				**< 0.001**	**< 0.001**	**< 0.001**	**< 0.001**	**< 0.001**	**< 0.001**	**< 0.001**	**< 0.001**	**< 0.001**	**< 0.001**	**0.001**	**< 0.001**	**< 0.001**	**< 0.001**	**< 0.001**	**< 0.001**	**< 0.001**	**< 0.001**	**0.001**
**Lymphocytes to monocytes ratio**					3.638	3.634	3.636	3.496	3.826	3.723	2.663	2.773	6.827	4.671	3.652	3.864	3.231	2.732	2.729	2.811	2.549	5.096
					0.056	0.057	0.057	0.062	**0.050**	0.054	0.103	0.096	**0.009**	**0.031**	0.056	**0.049**	0.072	0.098	0.099	0.094	0.110	**0.024**
**Neuthophils**						0.011								0.358							0.165	0.616
						0.915								0.550							0.685	0.433
**Lymphocytes**							0.007							0.344							0.159	0.601
							0.931							0.557							0.690	0.438
**White bread consumption**								0.346						0.013							0.630	0.179
								0.556						0.909							0.427	0.673
**Alchool consumption**									2.051					1.345							0.476	0.937
									0.152					0.246							0.490	0.333
**Fibrinogen**										1.972				2.584							0.514	0.947
										0.160				0.108							0.473	0.331
**LDL-Cholesterol**											2.699			0.962							1.397	0.938
											0.100			0.327							0.237	0.333
**Triglycerides**												0.225		0.019							0.371	0.180
												0.635		0.890							0.542	0.672
**Relative DNMT1 expression**													4.086	4.369								3.209
													**0.043**	**0.037**								0.073
**BMI**															7.329						6.073	5.417
															**0.007**						**0.014**	**0.020**
**Glycated haemoglobin (HbA1c)**																0.174					4.108	5.145
																0.676					**0.043**	**0.023**
**French fries consumption**																	8.602				6.698	5.804
																	**0.003**				**0.010**	**0.016**
**Cholesterol**																		0.829			0.294	0.227
																		0.362			0.588	0.633
**Glucose**																			2.462		4.540	5.262
																			0.117		**0.033**	**0.022**
**Free fatty acids**																				0.056	0.304	0.088
																				0.812	0.581	0.767

Legends to supplementary tables

Table S1

Subtelomeric DNA methylation in the RASIG population stratified by age

*KW test: nonparametric comparison by the Kruskal–Wallis test of the CpG methylation level between age groups. Data are reported as median and interquartile range (IQ). Pairwise comparisons, adjusted for multiple comparisons, were performed by the Mann–Whitney U-test (comparisons with $p < 0.05$ are marked by the associated superscripts).

†GLM test: groups comparison by generalized linear model analysis. The model included the effects of sex, recruitment centre and age as covariates (*p* values refer to age groups). Bold text indicates a statistically significant *p* value. Definition of abbreviations is provided in the supplementary list.

Table S2

Correlation between subtelomeric DNA methylation and age in the RASIG population

*The confidence intervals for both Pearson (parametric) and Spearman (non-parametric) correlation coefficients are based on 1000 bootstrap re-samples. Stratification was by sex and recruitment centre. Bold text indicates a statistically significant *p* value.

Table S3

Comparison of different linear regression models for subtelomeric DNA methylation to age in the RASIG population

Bold text indicates a statistically significant *p* value.

Table S4

Linear regression model

*The standard errors and confidence intervals for Pearson correlation coefficients are based on 1000 bootstrap re-samples. Stratification was by sex and recruitment centre. Bold text indicates a statistically significant *p* value.

Table S5

MAST in age-stratified RASIG, GO, SGO and DS

§KW test: nonparametric comparison by the Kruskal–Wallis test of MAST between age groups (RASIG) and between subject groups (RASIG, GO, SGO and DS). Data are reported as median and interquartile range (IQ). Pairwise comparisons, adjusted for multiple comparisons, were performed by the Mann–Whitney U-test (comparisons with $p < 0.05$ are marked by the associated superscripts). Bold text indicates a statistically significant *p* value.

*Age range was 55-75 years.

†Age range was 35-54 years.

Table S6

Correlation between MAST and telomere length in RASIG

Confidence intervals for correlation coefficients are based on 1000 bootstrap re-samples. Stratification was by sex and recruitment centre (MAST *vs* age and telomere length *vs* age) and by sex recruitment centre and age (MAST *vs* telomere length). Bold text indicates a statistically significant *p* value.

Table S7

Effect of recruitment centre, sex and BMI on MAST in RASIG

*KW test: nonparametric comparison by the Kruskal–Wallis test of MAST level between sample groups categorized by recruitment centre, sex and BMI. Data are reported as median and interquartile range (IQ). Pairwise comparisons, adjusted for multiple comparisons, were performed by the Mann–Whitney U-test (comparisons with $p < 0.05$ are marked by the associated superscripts).

†GLM test: groups comparison by generalized linear model analysis. The models included the effects of sex (§), recruitment centre (‡) or both (ə) as covariates (*p* values refer to the categories). Bold text indicates a statistically significant *p* value. Definition of abbreviations is provided in the supplementary list.

Table S8

Influence of dietary habits and lifestyle on MAST in RASIG

*KW test: nonparametric comparison by the Kruskal–Wallis test of MAST level between sample groups categorized by the indicated factor intervals. Data are reported as median and interquartile range (IQ). Pairwise comparisons, adjusted for multiple comparisons, were performed by the Mann–Whitney U-test (comparisons with $p < 0.05$ are marked by the associated superscripts).

†GLM test: groups comparison by generalized linear model analysis. The models included the effects of sex and recruitment centre as covariates (*p* values refer to the categories). Data were obtained after questionnaires as described in Moreno-Villanueva et al. [33]. Bold text indicates a statistically significant *p* value.

Table S9

Influence of cardiovascular and diabetes risk biomarkers on MAST in RASIG

*KW test: nonparametric comparison by the Kruskal–Wallis test of MAST level between sample groups categorized by the indicated variable intervals. Data are reported as median and interquartile range (IQ). Pairwise comparisons, adjusted for multiple comparisons, were performed by the Mann–Whitney U-test (comparisons with $p < 0.05$ are marked by the associated superscripts).

†GLM test: groups comparison by generalized linear model analysis. The models included the effects of sex and recruitment centre as covariates (*p* values refer to the categories). Bold text indicates a statistically significant *p* value. Definition of abbreviations is provided in the supplementary list. Details about the analytic procedures for the measurement of the parameters are given in Moreno-Villanueva et al. [32].

Table S10

Influence of the expression of DNA methylation machinery components on MAST in RASIG

*KW test: nonparametric comparison by the Kruskal–Wallis test of MAST level between sample groups categorized by the indicated variable intervals. Data are reported as median and interquartile range (IQ). Pairwise comparisons, adjusted for multiple comparisons, were performed by the Mann–Whitney U-test (comparisons with $p < 0.05$ are marked by the associated superscripts).

†GLM test: groups comparison by generalized linear model analysis. The models included the effects of sex and recruitment centre as covariates (*p* values refer to the categories). Bold text indicates a statistically significant *p* value. Definition of abbreviations is provided in the supplementary list. Details about the analytic procedures for the measurement of the parameters are given in Ciccarone et al. [36] and in Valentini et al. [67].

Table S11

Influence of haematological factors on MAST in RASIG

*KW test: nonparametric comparison by the Kruskal–Wallis test of MAST level between sample groups categorized by the indicated variable intervals. Data are reported as median and interquartile range (IQ). Pairwise comparisons, adjusted for multiple comparisons, were performed by the Mann–Whitney U-test (comparisons with $p < 0.05$ are marked by the associated superscripts).
†GLM test: groups comparison by generalized linear model analysis. The models included the effects of sex and recruitment centre as covariates (*p* values refer to the categories). Bold text indicates a statistically significant *p* value. Definition of abbreviations is provided in the supplementary list. Details about the analytic procedures for the measurement of the parameters are given in Moreno-Villanueva et al. [32].

Table S12
Contribution of selected variables and covariates on age-related changes of MAST in RASIG
Models: MAST (s), response variable; age group (o), factor variable; sex (n), recruitment centre (n), white bread consumption (s), alcohol consumption (s), triglycerides (s), LDL cholesterol (s), lymphocytes (s), ratio of lymphocyte to monocyte (s), neutrophils (s), fibrinogen (s), DNMT1 expression (s), BMI (s), French fries consumption (s), glycated haemoglobin (s), cholesterol (s), serum glucose (s) and free fatty acids (s) were included as covariates (o = ordinal variable; n = nominal variable; s = scale variable). Bold text indicates a statistically significant *p* value.

Table S13
Contribution of selected variables and covariates on group-related (GO, SGO and RASIG) changes of MAST in individuals aged > 54 years.
Models: MAST (s), response variable; subject group (o), factor variable; age (s), sex (n), recruitment centre (n), white bread consumption (s), alcohol consumption (s), triglycerides (s), LDL cholesterol (s), lymphocytes (s), ratio of lymphocyte to monocyte (s), neutrophils (s), fibrinogen (s), DNMT1 expression (s), BMI (s), French fries consumption (s), glycated haemoglobin (s), cholesterol (s), serum glucose (s) and free fatty acids (s) were included as covariates (o = ordinal variable; n = nominal variable; s = scale variable).
